# Intraoperative Bioprinting for Craniomaxillofacial Bone Reconstruction in Rats and Sheep

**DOI:** 10.1002/smsc.202400621

**Published:** 2025-09-09

**Authors:** Miji Yeo, Deepak Gupta, Irem Deniz Derman, Sendegul Yildirim, Yogendra P. Singh, Ethan Michael Gerhard, Elias Rizk, Thomas Neuberger, Scott Simon, Ibrahim T. Ozbolat

**Affiliations:** ^1^ Engineering Science and Mechanics Pennsylvania State University University Park PA 16802 USA; ^2^ Huck Institutes of the Life Sciences Pennsylvania State University University Park PA 16802 USA; ^3^ Department of Histology and Embryology School of Medicine Akdeniz University 07070 Antalya Turkey; ^4^ School of Healthcare Science and Engineering Vellore Institute of Technology Vellore Tamil Nadu 632014 India; ^5^ Department of Biomedical Engineering Penn State University University Park PA 16802 USA; ^6^ Department of Neurosurgery College of Medicine Penn State University Hershey PA 17033 USA; ^7^ Materials Research Institute Pennsylvania State University University Park PA 16802 USA; ^8^ Penn State Cancer Institute Penn State University Hershey PA 17033 USA

**Keywords:** bone regeneration, intraoperative bioprinting, rats, sheep

## Abstract

Craniomaxillofacial reconstruction is challenging due to the requirement for diverse manual surgical interventions, which significantly increase as the defect volume enlarges. To address these concerns, we utilized intraoperative bioprinting (IOB) to reconstruct cranial bone defects in surgical settings. We formulated an innovative collagen‐based bioink supplemented with human adipose‐derived stem cells (hADSCs) or bone morphogenetic protein‐2 (BMP‐2). The concentration and dispersion state of collagen along with hADSCs were precisely adjusted to enhance cytocompatibility, bioprintability, and osteogenic activities. IOB was first performed via a 3‐axis bioprinter on a rat model having a critical‐sized calvarial defect (39.3 mm^3^), which was infilled within ≈30 s and resulted in ≈90% bone coverage area in 8 weeks. Secondly, IOB was conducted on sheep calvarial defects (1,209 mm^3^, ≈31‐fold larger compared to the rat defects) using a 6‐axis robotic arm, where IOB took ≈5 min per defect. On Week 12, sheep defects treated with IOB revealed accelerated bone repair (≈80% bone coverage area) and mechanical enhancement with 240%, 235%, and 358% increments in Young's modulus, peak force, and energy compared to the non‐treated group. The successful execution of IOB in small and large animal models validates the translation potential of IOB for automated surgical interventions.

## Graphical abstract

In rat and sheep calvarial models, a highly concentrated collagen‐based bioink (HC‐ink) is bioprinted and compared in combination with recombinant human bone morphogenetic protein‐2 (rhBMP‐2) or human adipose‐derived stem cells. Collectively, HC‐ink with the highest cell density and the addition of rhBMP‐2 contributes to improved new bone formation in rat and sheep models, respectively.

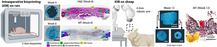

## 1 Introduction

Craniomaxillofacial (CMF) defects, typically involving large and complex bone damages, pose a significant reconstructive challenge due to their size and the critical need for precise anatomical restoration. Each year, over 7 million new cases of CMF are reported globally.^[^ ^1^ ^]^ These defects, arising from congenital deformities, trauma, tumors, infections, or conditions like stroke, often require prompt and effective surgical interventions to restore both function and aesthetics. Conventional reconstruction techniques, such as cranioplasty, typically rely on autologous, allogeneic, or synthetic bone grafts composed of polymers or ceramics.^[^ ^2^, ^3^, ^4^, ^5^ ^]^ Autografts are often considered the gold standard due to their excellent osteointegration, osteoconductivity, and mechanical compatibility with the native bone.^[^ ^3^, ^6^ ^]^ However, autologous grafts are limited by donor site morbidity, restricted availability, and the necessity for additional surgical procedures for harvesting and shaping.^[^ ^7^ ^]^ Allografts offer greater availability but come with inherent risks such as disease transmission, immune rejection, and infection and may also exhibit biological and structural incompatibilities that can hinder their effectiveness.^[^ ^8^ ^]^ While synthetic bone grafts allow for some degree of customization, they face significant challenges, including insufficient control over the hard/soft tissue interface, inadequate osteoconductivity, and suboptimal mechanical strength, all of which severely limit their clinical feasibility.^[^ ^7^ ^]^

Recent advancements in 3D bioprinting, particularly in situ or intraoperative bioprinting (IOB), have enhanced the development of defect‐specific biomimetic bone grafts. IOB enables the direct deposition of bioinks at the defect site during a surgery, allowing for the fabrication of anatomically relevant constructs. This approach can seamlessly integrate defect imaging with on‐site fabrication, reducing the need for extensive surgical interventions.^[^ ^9^ ^]^ IOB facilitates real‐time adjustments, eliminating the need for postprocessing implant modifications. The feasibility and potential of IOB for CMF reconstruction have been demonstrated in small animal models. For instance, laser‐assisted bioprinting (LAB) has been used to organize endothelial cells in situ within a mouse calvarial defect prefilled with collagen, mesenchymal stem cells, and vascular endothelial growth factor, effectively promoting prevascularization and bone regeneration through controlled in vivo cell patterning.^[^ ^10^ ^]^ However, the complex setup and slower processing speeds associated with LAB make it less practical for clinical translation. Extrusion‐based bioprinting (EBB) is a more clinically viable alternative due to its rapid operation, versatility, and compatibility with a broad range of bioinks, making it suitable for repairing large defects. For example, Li et al. utilized a robotic manipulator for IOB in swine tibia defects, optimizing bioink gelation to achieve appropriate mechanical properties and enhancing printing accuracy to 0.5 mm, which facilitated effective defect repair.^[^ ^11^ ^]^ However, challenges such as nozzle clogging and the requirement for dual crosslinking using calcium ions and ultraviolet (UV) light were noted. The ionic crosslinking process can react with blood at the defect site, aggravating nozzle clogging issues.^[^ ^12^ ^]^ To address these limitations, thermal crosslinking is a suitable alternative for bioinks, offering biocompatibility under physiological conditions without the need for harmful UV light or toxic agents. It provides controlled gelation, enhanced structural stability, and compatibility with natural polymers like collagen.^[^ ^13^ ^]^ Moreover, it is less susceptible to local environmental factors, thereby reducing the risks of clogging and inconsistent gelation.

The selection of suitable bioinks is vital for the success of IOB. These materials must exhibit biocompatibility, mechanical integrity, and suitability for point‐of‐care applications in surgical settings. In our previous work, we developed a collagen‐based thermally crosslinkable hydrogel, termed “hard tissue ink (HT‐ink),” for IOB into rat calvarial defects.^[^ ^14^ ^]^ Although HT‐ink demonstrated high biocompatibility, the incorporation of live cells significantly altered its rheological properties, leading to poor performance in vivo.^[^ ^15^ ^]^ We hypothesize that this was due to dilution effects and cell‐induced collagenase activity, which accelerated collagen degradation,^[^ ^16^ ^]^ rendering it unsuitable for bone regeneration. To overcome these limitations, we have developed a highly concentrated collagen‐based bioink (HC‐ink), which enhanced the homogeneity and stability of the bioink that is crucial for maintaining consistent printability and preserving the structural integrity of bioprinted constructs. Additionally, the thermal crosslinking of the bioink provides further advantages under physiological conditions, such as providing safe crosslinking, eliminating the need for additional crosslinking chemicals, and reducing nozzle clogging caused by ionic or enzymatic crosslinking.^[^ ^12^ ^]^

**Figure 1:**
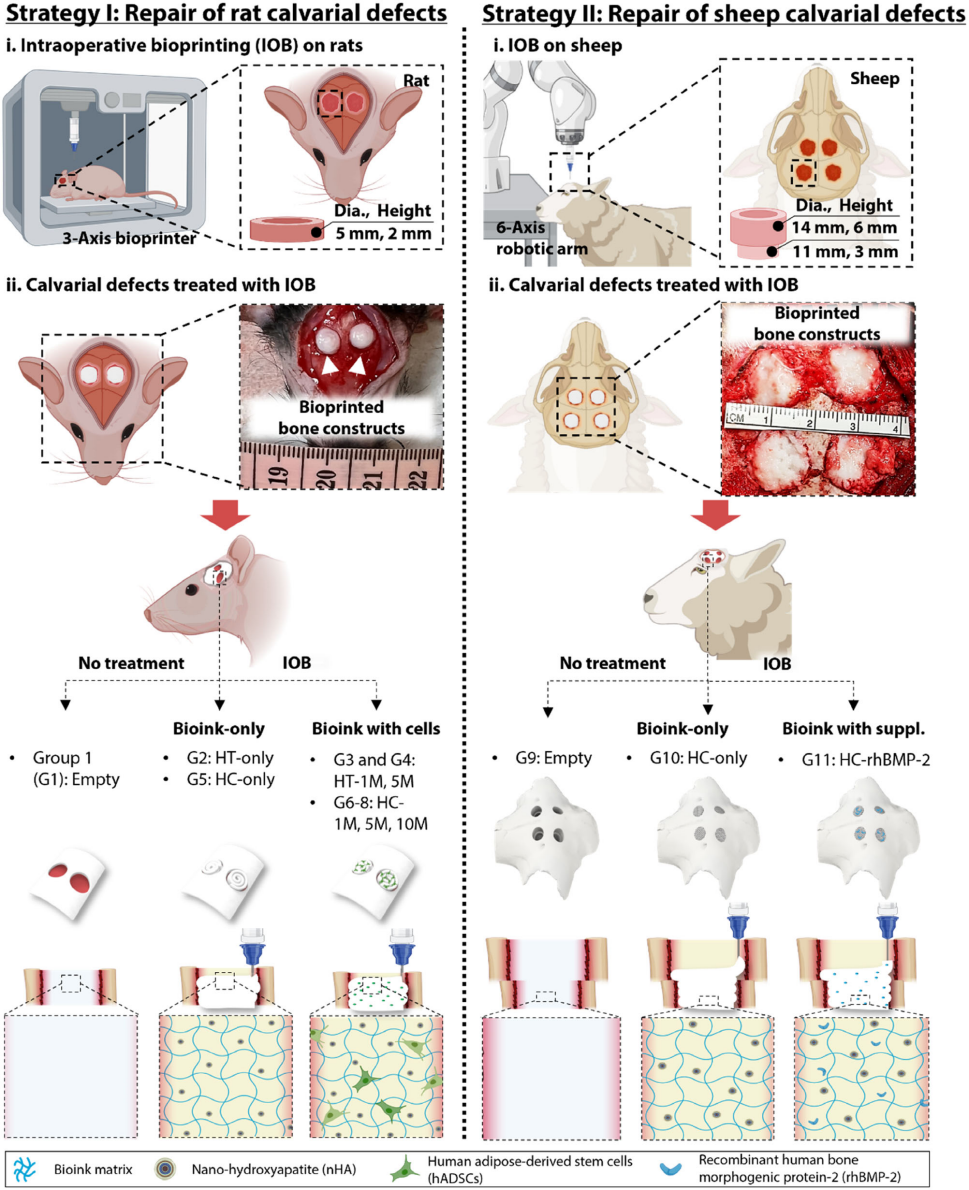
Schematic illustration of IOB on rats and sheep. Implementation of IOB for rat calvarial defect reconstruction using the HT‐ink (without cells, with 1 and 5 M hADSCs mL^−1^) and the HC‐ink (without cells, with 1, 5, and 10 M hADSCs mL^−1^). Likewise, IOB was implemented for sheep calvarial defect reconstruction, where the empty (control), HC‐only, and HC‐ink loaded with rhBMP‐2 (30 μg per defect) groups were tested.

In this study, we utilized the IOB technology and demonstrated its ability to generate tissue‐specific CMF implants using both a 3‐axis bioprinter and a 6‐axis robotic arm yielding notable regenerative capacity in both rat and sheep calvarial defect models. We developed a bioink (HC‐ink) that was compatible with IOB and can be used in both acellular and cell‐laden forms. The efficacy of HC‐ink was benchmarked against the previously developed HT‐ink, demonstrating fourfold greater in storage modulus enabling the loading of human adipose‐derived stem cells (hADSCs) at concentrations up to 10M cells/mL. IOB was first performed for critical‐sized calvarial defects in rats using a 3‐axis bioprinter, followed by calvarial defects in sheep using a 6‐axis robotic arm (**Figure** **1**). This study provides one of the most comprehensive analyses of IOB in both small and large animal models, particularly effective for addressing large and irregular defects like those in cranial perforators with intricate geometries. The findings demonstrate the successful incorporation of cells in the new HC‐ink, the scalability of the process from a small to a large animal model, and the real‐time fabrication of customized bone constructs. This study holds the potential to significantly advance the IOB technology for the treatment of complex and substantial bone defects.

## 2 Results

### 2.1 Physical Properties and Biocompatibility of the HT‐ and HC‐Inks

Physical characterization of the bioinks was conducted to conform their suitability for bioprinting and in vivo applications for bone regeneration. Both HT‐ and HC‐inks were formulated from chitosan, β‐Glycerophosphate disodium salt hydrate (β‐GP), nanohydroxyapatite powder (nHAp), and collagen (Figure S1A, Supporting Information). However, the HC‐ink contained higher collagen concentration of 66 mg mL^−1^ in dissolved form as compared to 40 mg mL^−1^ present in the HT‐ink in dispersed form. Individual components were confirmed by characteristic peaks via the attenuated total reflection–Fourier‐transform infrared spectroscopy (Figure S2A, Supporting Information). Briefly, the amide I (1650 cm^−1^) and II (1550 cm^−1^) bands and internal vibration of PO_4_ (900–1200 cm^−1^ and 500–620 cm^−1^) were identified for Col1 and nHA, respectively.^[^ ^17^, ^18^ ^]^ Both HT‐ink (*P*_r_: 1.2 ± 0.3) and HC‐ink (*P*_r_:1.1 ± 0.1) demonstrated reasonable printability (Figure S2B, Supporting Information), suitable for replicating the concentric architecture of the native rat calvarial bone. Young's moduli were comparable between HT‐ink (5 ± 0.2 kPa) and HC‐ink (6 ± 1 kPa) (Figure S2C, Supporting Information). When swelling was assessed using manually deposited (HT‐ink‐M and HC‐ink‐M) or printed (HT‐ink‐P and HC‐ink‐P) constructs, the HC‐ink group absorbed liquid for 1 h and reached plateau, while erosive effects were more evident in the HT‐ink group (Figure S2D, Supporting Information). Degradation behavior was observed using DPBS and collagenase for 8 weeks (Figure S2E,F, Supporting Information). Under DPBS at 37 °C, the HT‐ink groups completely degraded over 8 weeks, whereas the HC‐ink groups retained ≈40% of the construct structure. This observation underscores the extended durability of the HC‐ink, attributed to its higher collagen concentration. The incorporation of cells is expected to further facilitate degradation, as supported by subsequent collagenase data. Therefore, hADSCs embedded in the HC‐ink are likely to sustain cellular activity and contribute to bone regeneration over an extended period. Moreover, in collagenase solution, both HT‐ and HC‐ink groups exhibited complete degradation by Week 4. Assuming similarity to in vivo physiological conditions, the remodeling of the bioink matrix is expected to be facilitated by cellular activity and host–tissue interaction in 4 weeks postimplantation. Meanwhile, no significant difference was found between manually loaded and bioprinted constructs.

To assess changes in rheological properties with the addition of cells, hADSCs were incorporated at 5 million (M) mL^−1^ into both the HT‐ and HC‐inks, referred to as HT‐5 M and HC‐5 M, respectively. Specifically, HC‐only exhibited higher viscosity (*η*), while both bioinks demonstrated shear‐thinning behavior, as evidenced by a decrease in viscosity with an increase in shear rate ($\overset{\cdot }{\gamma }$) (Figure S1B, Supporting Information). The amplitude sweep of the bioinks revealed a plateau region at lower strains, with the storage modulus (*G′*) dominating over the loss modulus (*G″*), indicating that the bioink exhibited solid‐like (elastic) behavior (Figure S1C, Supporting Information). It was also observed that the HC‐only had a significantly higher *G′* (1,207 Pa) than that of the HT‐only (352 Pa), indicating its higher elastic strength. In addition, *G′* of the HT‐ and HC‐ink decreased to 174 Pa and 627 Pa, respectively, after the addition of 5M hADSCs/mL (Figure S1D, Supporting Information). The results in Figure S1C, Supporting Information, also demonstrated that *G′* of all bioinks decreased at higher strain values, and the bioinks yielded at their respective *G′*–*G″* crossover (*G′* = *G″*) points, confirming that they exhibited yield stress behavior. The bioinks transitioned from solid‐like to liquid‐like state at their yield stress points, after which *G″* exceeded *G′* with an increase in strain mimicking the extrusion step during bioprinting. It was found that the yield stress of HC‐only (1.1 Pa) was significantly higher than that of HT‐only (0.5 Pa). The yield stress of both HT‐ and HC‐only decreased by ≈60% and ≈85%, respectively, after adding 5 M hADSCs mL^−1^; however, HC‐5 M showed significantly higher *G′* than HT‐5 M (Figure S1D, Supporting Information). This indicates that dissolved collagen in the HC‐ink made it more homogeneous with enhanced gel‐like properties, thus offering consistency and better structural stability during bioprinting. To evaluate the time‐dependent behavior of the bioinks within the linear‐viscoelastic range, frequency sweep tests were conducted. The results showed that *G′* was higher than *G″,* indicating that HT‐ and HC‐only remained in the stable elastic region at all frequencies (Figure S1E, Supporting Information). Here, HC‐only yielded significantly higher values than HT‐only. On the other hand, the decreasing complex viscosity (η*) over increasing frequency confirmed the pseudoplasticity of bioinks, making it favorable for EBB (Figure S1E, Supporting Information).

In this study, hADSCs were added into the bioinks to improve bone tissue regeneration; however, this also diluted and altered rheological properties of the bioinks, making it necessary to perform their bioprintability characterization. Hence, a continuous extrusion test was carried out with different bioprinting conditions to form lattice‐shaped structures (Videos S1 and S2, Supporting Information). The targeted filament diameter was set to 500 μm using 80–140 kPa pneumatic pressure and 22 G extrusion nozzle (Table S1, Supporting Information). The diameter of filaments bioprinted with different bioinks was determined to be 470.8 ± 14.4 μm, averaged across all groups; no significant difference was observed among the groups (Table S2, Supporting Information). Stable extrusion with continuous filament formation and uniform diameter was achieved with the HT‐ink containing 1 and 5 M hADSCs, but not with 10 M hADSCs. The excess dilution at 10 M hADSCs decreased the bioink's viscosity and deteriorated its bioprintability. On the other hand, the HC‐ink maintained reasonable bioprintability even with the addition of 10 M hADSCs, thus validating its suitability for bioprinting. Therefore, we opted for HT‐1 M and −5 M as well as HC‐1 M, −5 M, and −10 M for further studies.

### 2.2 Cytocompatibility and Osteogenesis of Bioprinted HADSCs

**Figure 2:**
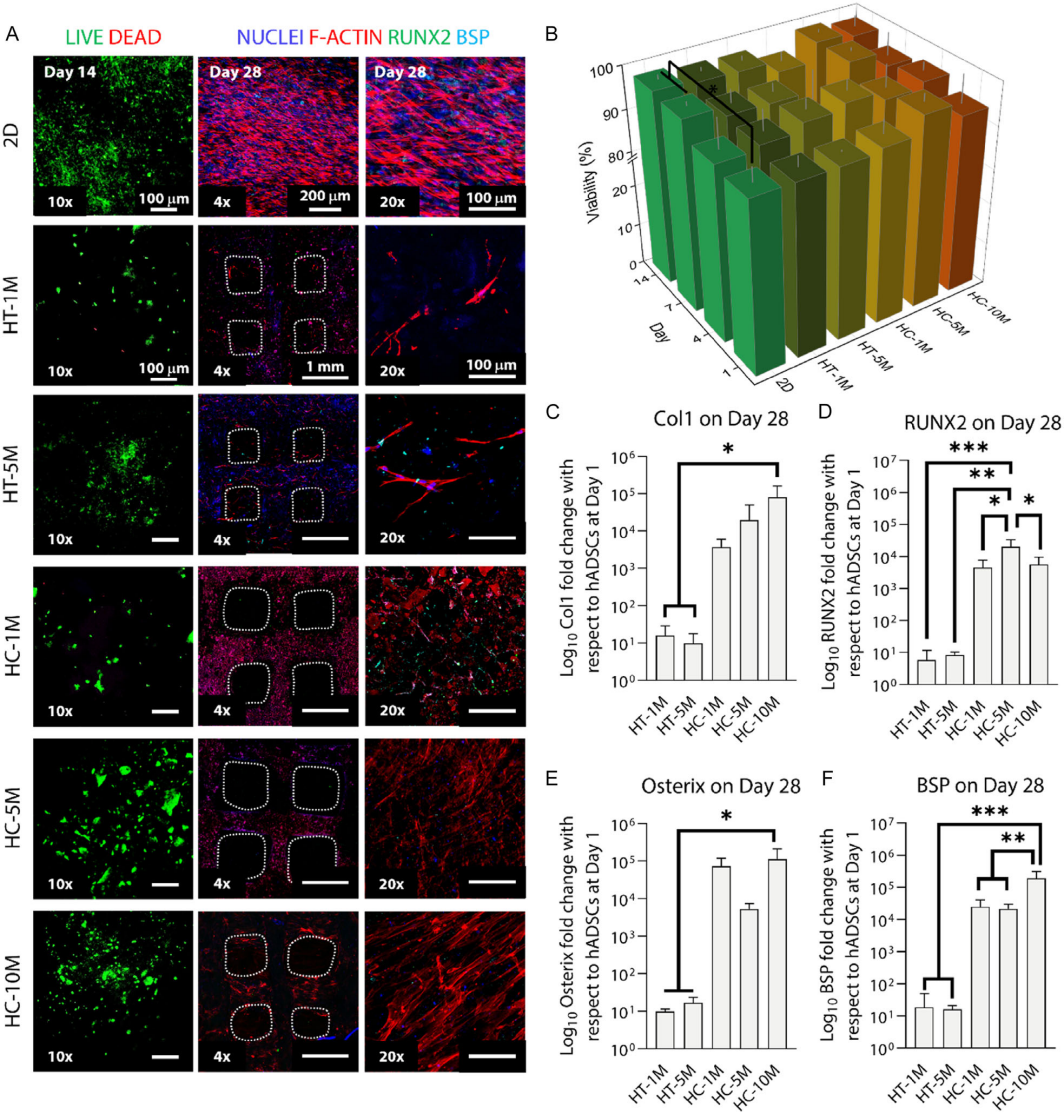
Evaluation of cytocompatibility and osteogenic behavior of hADSCs in HT‐ and HC‐ink constructs. A) LIVE/DEAD staining for observing live (green) and dead (red) cells to study cytocompatibility on Day 14. Immunostaining of hADSCs detecting the commitment of osteogenic cells (RUNX2 in green) and late‐stage osteogenesis (BSP in cyan), counterstained with nuclei (DAPI in blue) and cytoskeletal structures (Phalloidin in red) on Day 28. B) Quantitative analysis of cell viability on Day 14 (*n* = 5). qRT‐PCR analysis of the HT‐ and HC‐inks loaded with varying densities of hADSCs, evaluated by the expression of osteogenic gene markers: C) Col1, D) RUNX2, E) Osterix, and F) BSP on Day 28 (*n* = 4). *p < 0.05, **p < 0.01, ***p < 0.001. ‘ns’ indicates no significant differences.

The cytocompatibility of bioinks was investigated by LIVE/DEAD staining. For this, cell‐laden bioinks were bioprinted into bulk constructs, and the results demonstrated >90% viability in all samples throughout the 14‐day culture (**Figure** **2**A,B and S3, Supporting Information). Additionally, cell viability increased over time. These results indicate that bioprinted constructs were cytocompatible and could be used for both in vitro and in vivo applications.

To evaluate osteogenic differentiation of hADSCs, cells were bioprinted in 3D lattice patterns at varying cell densities, resulting in 0.9 × 10^5^, 4.4 × 10^5^, and 8.8 × 10^5^ cells/construct for bioinks with 1, 5, and 10 M hADSCs mL^−1^, respectively. The constructs were visualized for RUNX2 (green), BSP (cyan), and Phalloidin (red) counterstained with DAPI (blue) on Days 14 and 28. RUNX2 serves as a transcription factor crucial for initiating osteogenic activity during the early stages of osteogenesis.^[^ ^19^ ^]^ On the other hand, BSP is highly expressed in mature bone, which can be used as an index of the deposited mineralized bone matrix.^[^ ^20^ ^]^ While acellular constructs exhibited no fluorescence signal (Figure S4A, Supporting Information), a decrease in RUNX2 expression and an enhancement of BSP expression were observed in HC‐ink constructs from Day 14 to 28 (Figure 2A and S4B, Supporting Information). Osteogenic gene markers were further assessed via quantitative real‐time polymerase chain reaction (qRT‐PCR) at Day 28 (Figure 2C–F). Collagen type I (Col1) is a major component of bone extracellular matrix and is another crucial early osteogenic marker that validates the osteogenic potential of cells.^[^ ^21^ ^]^ Osterix is a transcription factor that plays a role in intramembranous bone formation and mineralization. All gene expressions—Col1, RUNX2, osterix, and BSP—were upregulated in the HC‐ink groups. Although RUNX2 is an early osteogenic marker, the sustained high levels observed here may reflect its active role in promoting osteoblastic differentiation, typically in the HC‐ink groups, by inducing downstream markers such as osterix and BSP.^[^ ^22^ ^]^ These findings imply that the HC‐ink was preferable for inducing osteogenic activities.

### 2.3 IOB of Bone Constructs into Rat Calvarial Defects

**Figure 3:**
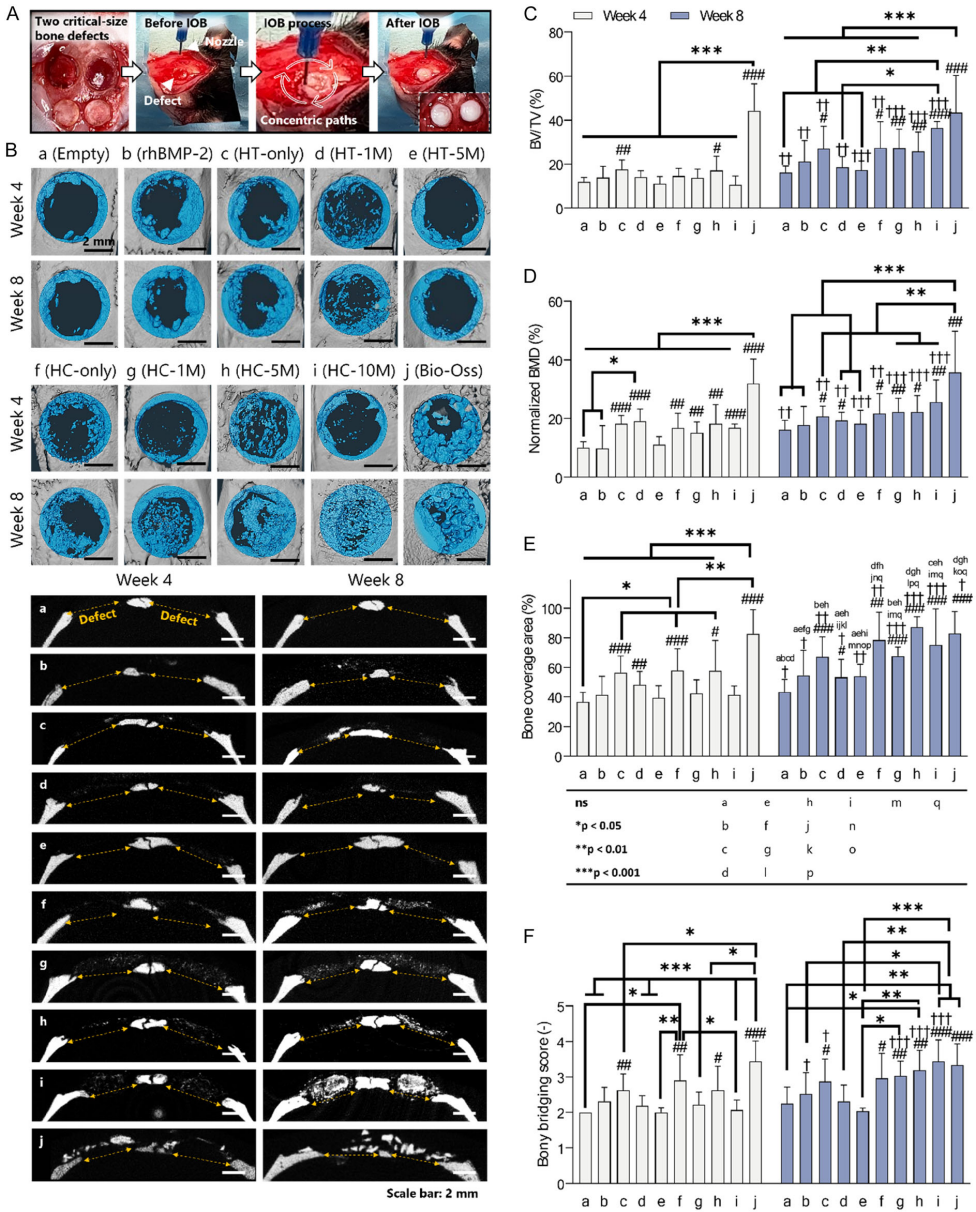
In vivo analysis of bone regeneration via μCT scanning 4 or 8 weeks after IOB into rat calvarial defects. A) Images demonstrating surgical procedures of IOB on rat calvarial defects. B) Representative μCT images showcasing newly formed bone in blue, filtered using a threshold exceeding 300 mmHg ccm to indicate hard bone. Quantitative analysis concerning C) BV/TV (%), reflecting the ratio of bone volume to total volume within the defect; D) normalized BMD (%), indicating BMD relative to the native bone; E) bone coverage area (%), representing the extent of the defect covered by the new bone; and F) bony bridging score, assessing the quality and extent of bone connection across the defect site (*n* = 8). # referred to the one‐on‐one comparison between the empty (control) and the experimental groups via independent‐samples T test (^#^ *p* < 0.05, ^##^ *p* < 0.01, ^###^ *p* < 0.001). † referred to the comparison between two time points (Week 4 and 8) within the same group via paired‐samples T test (^†^ *p* < 0.05, ^††^ *p* < 0.01, ^†††^ *p* < 0.001). * referred to the comparison among the HT‐ and HC‐ink groups at Week 4 or 8 via one‐way ANOVA (*p < 0.05, **p < 0.01, ***p < 0.001).

Following a comprehensive characterization of the HC‐ink in vitro, IOB was performed under surgical settings. For this, two critical‐sized calvarial defects (5 mm in dia. and 2 mm in thickness) were surgically created on rat skulls (**Figure** **3**A). A 3‐axis bioprinter was used to bioprint anatomically accurate bone constructs directly into the defects using bioinks at various cell densities (Table S3, Supporting Information). Cylindrical constructs were bioprinted, where the bioprinter head followed a spiral path with a 100% infill density in ≈30 s per construct (Video S3, Supporting Information). In this set of animal experiments, 10 groups were investigated including empty (control), the HC‐ink with rhBMP‐2, the HT‐ink with 0, 1, and 5 M hADSCs mL^−1^, the HC‐ink with 0, 1, 5, and 10 M hADSCs mL^−1^, and Bio‐Oss. Postsurgery, defect sites were assessed with in vivo live micro computed tomography (μCT) scanning at Week 4 and the rats were sacrificed for further evaluation at Week 8.

Representative μCT images depicted regenerated hard bone (300 mgHA ccm) in blue color after 4‐ and 8‐weeks post‐IOB (Figure 3B). The gross images suggest a progressive increase in bone growth from the empty to HC‐ink groups and from Week 4 to 8. The percentage of new bone volume to total bone volume (BV/TV) in both Weeks 4 and 8 showed that the incorporation of hADSCs in the HT‐ink was not conducive for new bone formation, characterized by a decreasing trend with higher cell density (Figure 3C). This could be attributed to rheological dilution and cell‐induced collagenase activities, which might lead to the rapid degradation of the HT‐ink. On the contrary, hADSCs improved bone formation in the HC‐ink due to the higher collagen content in dissolved form. While Bio‐Oss demonstrated the highest regenerated bone volume, rhBMP‐2 did not differ from either the HT‐ or HC‐ink. Similarly, normalized bone mineral density (BMD) resulted in the highest value for the Bio‐Oss group at Weeks 4 and 8. However, a gradual increment in BMD was observed over time, especially for the HC‐ink groups (Figure 3D). The bone coverage area (%) significantly increased in the HT‐ink groups except the HT‐5 M group and all HC‐ink groups compared to the empty group (Figure 3E). Interestingly, cell‐laden HC‐ink groups displayed homogeneously dispersed HT fragments throughout the defects at Week 8 (Figure S5 and S7, Supporting Information). However, the nonspecific particle distribution was observed with the Bio‐Oss group, and the HT‐ink with hADSCs exhibited decreasing bone coverage area in accordance with BV/TV results. The bony bridging score was assessed using the μCT datasets, which serve as an index to score regenerated bone morphology from 0 (no bone formation) to 4 (bony bridging of entire span at longest point (5 mm)).^[^ ^23^ ^]^ As a result, the HC‐10 M revealed the highest score (3.4 ± 0.6), which was significantly higher than the empty (2.3 ± 0.5) or HT‐5 M (2.1 ± 0.2) groups (Figure 3F). These results demonstrated that increasing cell density in the HC‐ink enhanced bone regeneration.

To confirm cell loading and fate postsurgery, hADSCs were transduced with Tandem‐dimer tomato (tdTomato; red) lentivirus and incorporated into the HC‐ink at 10 M mL^−1^ (Figure S6A, Supporting Information). After 2 weeks of IOB, rat calvaria was harvested and stained for RUNX2 (green) to assess bone differentiation. Notably, tdTomato^+^ hADSCs appeared sparsely distributed across the defect, with a gradual overlap of red and green fluorescence suggesting the transition of hADSCs into osteogenic lineage. Likewise, histological staining also depicts the progress of bone regeneration in the defect site (Figure S6B, Supporting Information). Briefly, hADSCs showed their potential to commit and contribute to osteogenesis over time. Indeed, tracking hADSCs up to mature bone remodeling is valuable to understand the role of exogenous hADSCs and distinguish them from endogenous cells involved in bone regeneration. However, as we acknowledged in our previous study,^[^ ^24^ ^]^ the fluorescence lifetime of tdTomato diminishes rapidly, likely due to factors like cell proliferation, mitotic events, and cell–cell interactions.^[^ ^25^ ^]^ Therefore, less abundant fluorescence can be comprehended due not only to cell death but also to metabolic activity, differentiation, and interactions with host tissues; these challenges should be addressed in future studies to enable sustained tracking during bone regeneration.

**Figure 4:**
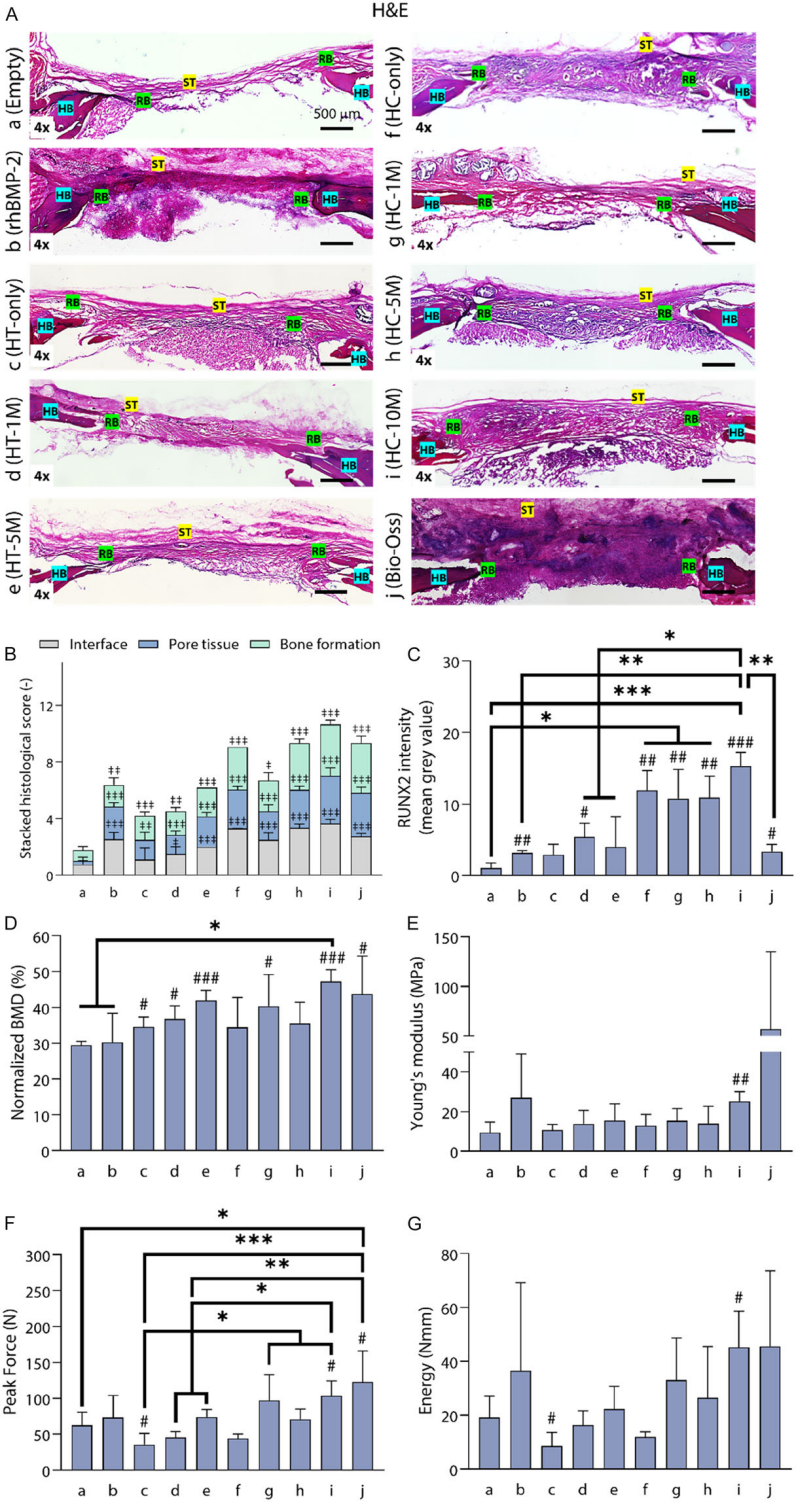
Histomorphometric and IHC analysis of rat calvarial defects at Week 8. A) H&E images demonstrating native bone (NB), regenerated bone (RB), and soft tissue (ST). B) Cumulative histological scoring was assessed by the quality of the interface (interface), cellular response in the defect region (pore tissue), and bone fraction in the defect region (bone formation). C) Quantitative measurement of RUNX2 intensity (*n* = 3). D) Normalized BMD of edge regions of rat calvarial defects (*n *= 4). The results of the push‐out test concerning E) stiffness (N mm^−1^), F) peak force (N), and G) energy (Nmm) (*n *= 4). # referred to the one‐on‐one comparison between the empty (control) and experimental groups via independent‐samples T test (^#^ *p* < 0.05, ^##^ *p* < 0.01, ^###^ *p* < 0.001). ‡ referred to the one‐on‐one comparison between the empty (control) and experimental groups for each histological scoring parameter (interface, pore tissue, and bone formation) via independent‐samples T test (^‡^ *p* < 0.05, ^‡‡^ *p* < 0.01, ^‡‡‡^ *p* < 0.001) * referred to the comparison among the HT‐ and HC‐ink groups via one‐way ANOVA (*p < 0.05, **p < 0.01, ***p < 0.001).

Bone regeneration was also analyzed histologically using hematoxylin and eosin (H&E) and Masson's trichrome staining (MTS) (**Figure** **4**A and S8A, Supporting Information). In H&E images, native bone (NB) exhibited intense eosin (red) staining compared to that of the regenerated bone (RB), while soft tissue (ST) was stained in pink. To elaborate, the empty group revealed a clear soft tissue bridging and new peripheral bones in some areas. Compared to the empty group, the HT‐ and HC‐ink groups exhibited higher RB below the ST layer. Corroborating with the μCT data, the HT‐ink groups illustrated thicker RB tissue as the cell density decreased. The HC‐ink demonstrated the formation of thicker and intensely red‐colored (indicative of greater maturity) bone structures at the highest cell density, with the initiation of bone fraction formation observed with cell densities exceeding 5 M cells mL^−1^. Meanwhile, the HC‐ink with rhBMP‐2 demonstrated bone regeneration comparable to the HC‐only and HC‐1 M groups. Among all groups, the Bio‐Oss group represented a thicker RB layer with an intense red color, although the RB extended beyond the defect site, indicating ectopic bone formation.^[^ ^26^ ^]^ Likewise, MTS, which stains collagen with a light blue color, illustrated immature bone formation in the empty, rhBMP‐2, and HT‐ink groups (Figure S8A, Supporting Information). In contrast, the increased tissue thickness observed in the HC‐ink and Bio‐Oss groups exhibited grayish or reddish staining, indicative of ongoing ossification.^[^ ^27^, ^28^ ^]^ Additionally, for a quantitative evaluation, histological scoring was performed (Figure 4B).^[^ ^23^ ^]^ The cumulative scores were obtained by evaluating the quality of the bone–scaffold interface (referred to as interface), bone or fibrous tissue formation and inflammatory response within the defect (referred to as pore tissue), and quantity of bone formation (referred to as bone formation). These parameters were scored from score 0 (lowest) to 4 (highest). Interestingly, cumulative scores of 6.4, 4.5, 6.2, and 9 were obtained for rhBMP‐2, HT‐only, ‐1 M, and ‐5 M, respectively. Here, some scoring parameters were similar to the empty group (4.2). Meanwhile, all the HC‐ink groups resulted in significantly improved histological scores, namely 9 for HC‐only, 6.7 for HC‐1 M, 9.3 for HC‐5 M, and 10.7 for HC‐10 M, while Bio‐Oss scored 9.3. In addition, bone formation was evaluated by immunohistochemistry (IHC) staining of the osteogenic marker (RUNX2) (Figure S8B, Supporting Information). RUNX2 is a representative marker in the early bone remodeling stage, which is actively involved in various processes like gathering of preosteoblasts, differentiation into osteoclasts, and matrix production.^[^ ^29^, ^30^ ^]^ Early osteogenic activities can be observed by abundant RUNX2 expression (Figure S7 and S8B, Supporting Information). Indeed, the RUNX2 expression was more visible in some groups, including HC‐1, ‐5, and 10 M, which were significantly higher compared to the empty group (Figure 4C). These IHC characterizations confirmed more effective bone regeneration of the HC‐ink with the addition of hADSCs.^[^ ^31^, ^32^ ^]^

Furthermore, mechanical strength was evaluated to assess neobone strength and functionality (Figure S9A, Supporting Information). The push‐out test data were analyzed to obtain normalized BMD, Young's modulus, peak force, and energy. The measurement of boundary BMD allowed us to obtain BMD specific to the hard bone, enabling the calculation of bone strength. As a result, HC‐10 M exhibited the highest BMD among the groups (Figure 4D). Young's modulus and peak force for HC‐10 M revealed the highest values, 2.7‐fold (Young's modulus) and 1.7‐fold (peak force), compared to the empty group (Figure 4E,F). In accordance with these properties, the energy absorption at the maximum load point was 2.4‐fold in HC‐10 M compared to the empty group (Figure 4G). The results of HC‐10 M did not statistically differ from those of the Bio‐Oss group, which revealed 6.0‐fold (Young's modulus), 2.0‐fold (peak force), and 2.4‐fold (energy) increases compared to the empty group. These findings indicate that the deposition of the HC‐ink with the highest cell concentration led to the formation of relatively hard bone. After the successful application of IOB for the reconstruction of calvarial defects in a small animal model, IOB was further validated on a larger animal model.

### 2.4 IOB of Bone Constructs into Sheep Calvarial Defects

**Figure 5:**
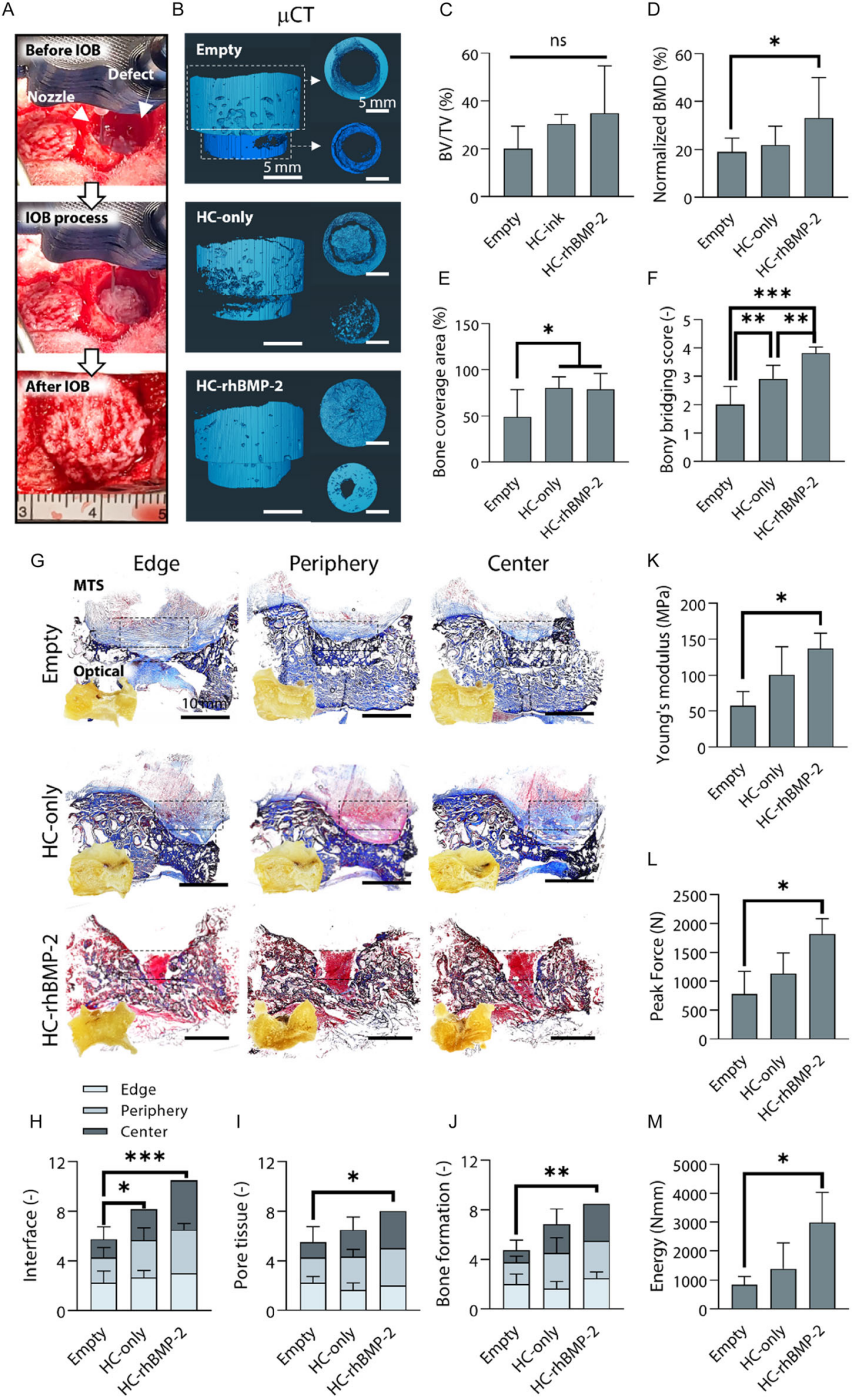
In vivo analysis of bone regeneration via μCT scanning and histomorphometric analysis 12 weeks after IOB into sheep calvarial defects. A) Optical images of the surgical setup and bioprinted constructs. B) μCT images showcasing hard bones in blue using a threshold exceeding 300 mmHg ccm^−1^. Quantitative analysis concerning C) BV/TV (%), D) normalized BMD (%), E) bone coverage area (%), and F) bony bridging score (*n* = 8). G) MTS images demonstrating blueish/greyish or reddish colors for the ongoing progress of ossification at different depth of defects from edge (≈3 mm) to center (≈9 mm). The histomorphometric characterization concerning H) interface, I) pore tissue, and J) bone formation. The results of push‐out test concerning K) stiffness (N mm^−1^), L) peak force (N), and M) energy (Nmm) (*n* = 4). * referred to the comparison among the empty (control), HC‐only, and HC‐rhBMP‐2 groups via one‐way ANOVA (*p < 0.05, **p < 0.01, ***p < 0.001).

The implementation of IOB in a sheep calvarial defect model was demonstrated via the use of a 6‐axis robotic arm. Cranial perforators having two compartments (upper compartment: 14 mm dia. and 6 mm thickness; lower compartment: 11 mm dia. and 3 mm thickness) were used to generate four defects on sheep skulls (**Figure** **5**A). The 6‐axis robotic arm operated at a reduced speed of 300 mm/min to ensure thorough filling of the two cylindrical compartments, where complete infilling was achieved within ≈5 min per defect. (Table S1 and Video S3, Supporting Information). The sheep were euthanized at Week 12 for the assessment of bone regeneration. We had three groups for the sheep study: empty, HC‐only, and the HC‐ink loaded with recombinant human BMP‐2 (HC‐rhBMP‐2) at 30 μg per defect. hADSCs were excluded to prevent a xenograft‐derived immune response, but rhBMP‐2, a clinically‐available growth factor,^[^ ^33^ ^]^ was added to support bone growth. Figure 5B and S10, Supporting Information, represents reconstructed μCT images, depicting regenerated bone in blue, with BMD exceeding 300 mmHg ccm^−1^. The empty group yielded bone regeneration only in peripheral regions, whereas the HC‐only and HC‐rhBMP‐2 groups showcased new bone formation in the central region as well. Although BV/TV did not differ significantly among the groups, the HC‐only and HC‐rhBMP‐2 groups showed a 1.5‐fold and 1.7‐fold increase in BV/TV, respectively, compared to the empty group (Figure 5C). Normalized BMD was enhanced in the HC‐rhBMP‐2 group (33 ± 17%), followed by the HC‐only group (22 ± 8%) and the empty group (19 ± 6%) (Figure 5D). Also, the bone coverage area reached ≈80% in bioprinted groups, while the empty group displayed ≈50% (Figure 5E). Finally, bony bridging score was assessed on a scale from 0 (no bone formation) to 4 (complete bridging of the entire span at the longest point). As expected, the HC‐rhBMP‐2 group revealed the highest score of 3.8 ± 0.2 while the HC‐only and empty group yielded 2.9 ± 0.5 and 2.0 ± 0.6, respectively (Figure 5F). These results demonstrated that the combination of HC‐ink and rhBMP‐2 significantly enhanced bone regeneration.

Histological analysis was also conducted at varying depths, corresponding to the defect thickness, which ranged from 3 mm at the edge to 9 mm at the central region (Figure S11, Supporting Information). H&E‐stained images revealed that the defect area increased in size toward the center. Porous or fibrous morphometric characteristics were found in the defect region for the empty and HC‐only groups. However, the defect area in HC‐rhBMP‐2 was distinctive as identified with densely packed structures. To further comprehend the regeneration process using MTS (Figure 5G), we observed a dense bony boundary on the surface of the defect in the empty group, which displayed an intense blue color. Meanwhile, a new bone structure was identified in the defect area of the HC‐only and HC‐rhBMP‐2 groups, showing a bluish‐reddish color, with blue indicating regenerated bone and red indicating mature bone (Figure 5G).^[^ ^34^ ^]^ Especially, the red region was dominant in the defect area of HC‐rhBMP‐2. In the process of bone remodeling, one can observe tightly packed but randomly organized bundles known as woven bone.^[^ ^35^ ^]^ These structures consist of disorderly arranged bundles of collagen fibers, numerous osteocytes, and irregular porous areas, where calcification or lacunar remodeling occurs.^[^ ^23^, ^35^ ^]^ Subsequently, MTS images exhibited similar morphometric profiles compared to those of H&E images. However, additional insights might be required for comprehensive understanding of bone maturity. Based on these, the HC‐rhBMP‐2 group revealed the most favorable condition for neobone and mature bone formation.

Moreover, qualitative and quantitative analysis were conducted via histology to interpret bone regeneration. Figure 5G visualizes MTS of the defect regions at different depths (edge, periphery, and center). The empty and HC‐rhBMP‐2 groups exhibited similar profiles, with defect regions enlarging in size without a distinct color change from the edge to the center, Conversely, the HC‐only group showed the transition of color from red to blue. To elaborate, the empty group constantly displayed a bluish defect region, indicating immature bone formation. In contrast, the HC‐only group showed bone maturation at the peripheral region with a red color, while the central area exhibited a red‐bluish blend, suggesting ongoing bone maturation. The addition of rhBMP‐2 to the HC‐ink accelerated bone maturation, as evidenced by the persistent red color observed in the HC‐rhBMP‐2 group. These results denote the positive effects of the HC‐ink on bone regeneration and demonstrate a significant improvement in efficacy with the introduction of bioactive cues, such as rhBMP‐2. Furthermore, the progress of bone formation was quantitatively assessed using a scoring system.^[^ ^23^ ^]^ The bone–scaffold interface, HT response within the pores of the scaffold, and bone formation within the defect were assessed.^[^ ^23^ ^]^ These were collectively represented from three regions (edge, periphery, and center) on a scale from 0 to 12. The bone–scaffold interface score was moderate for all groups since the biocompatibility and adequate mechanical strength of the bioink incited neither an immune response nor excessive shear stress^[^ ^36^ ^]^ (Figure 5H). The average score was 1.9 ± 0.9 for the empty group, 2.7 ± 0.6 for the HT‐only group, and 3.5 ± 0.5 for the HC‐rhBMP‐2 group. Although fibrous tissue formation or lacunar remodeling did not extend throughout the entire area, remodeling progress was observed in both HC‐only and HC‐rhBMP‐2 groups. The pore tissue score indicates the composition of tissue, such as bone, fibrous tissues, or inflammatory cells. For pore tissue characterization, the highest score 4 means the structure with mostly bone, while the lowest score 0 means the tissue filled with inflammatory cells without the presence of bone. The mean value of pore tissue was 1.8 ± 0.8, 2.2 ± 0.8, and 2.7 ± 0.5 for the empty, HC‐only, and HC‐rhBMP‐2 groups, respectively (Figure 5I). The HC‐rhBMP‐2 revealed the highest score as it was filled with immature bone or fibrous tissue while the defect area remained mostly fibrous for the other groups. Lastly, bone formation was scored from 0 (0% bone volume) to 4 (75–100% bone volume) with an incremental step of 25% (Figure 5J). The score was 1.6 ± 0.8, 2.3 ± 1.1, and 2.8 ± 0.4 for the empty, HC‐only, and HC‐rhBMP‐2 group, respectively. It is consistent with previous data that HC‐rhBMP‐2 provided a microenvironment that was favorable for new bone formation and maturation. The histomorphometric assessment indicates that HC‐rhBMP‐2 was promising for bone regeneration, which was further assessed via mechanical testing.

The mechanical testing setup was developed with a probe (dia.: 10 mm) and a plate with a hole (dia.: 12.5 mm) designed specifically for sheep calvarial defects (upper compartment dia.: 14 mm; lower compartment dia.: 11 mm) (Figure S9B, Supporting Information). For Young's modulus and peak force, the HC‐rhBMP‐2 group (137 ± 21 MPa and 1,819 ± 266 N) displayed a significant difference with respect to the empty group (57 ± 20 MPa and 774 ± 396 N) (Figure 5K,L). Meanwhile, the HC‐only group demonstrated 101.0 ± 38.6 MPa and 1,129 ± 362 N, which did not significantly differ from the empty or HC‐rhBMP‐2 groups. Also, the HC‐rhBMP‐2 group showed the highest load‐bearing capacity (2,988 ± 1,046 Nmm) by tolerating more energy, which was 3.6‐ and 2.1‐fold compared to the empty (1,394 ± 886 Nmm) and HC‐only (835 ± 288 Nmm) group, respectively (Figure 5M). These results highlight that the HC‐ink supplemented with rhBMP‐2 did not only facilitate bone regeneration but also improved the mechanical properties of the regenerated bone.

## 3 Discussion

The success of IOB for calvarial bone regeneration largely relies upon the effectiveness of bioinks, including their biocompatibility, processability, osteoinductivity, osteoconductivity, and osteointegrity. For IOB approaches, GelMA^[^ ^11^, ^37^ ^]^ and collagen at low concentrations (i.e., 2 mg mL^−1^)^[^ ^10^ ^]^ were introduced, but the use of UV light for crosslinking or low mechanical strength raised concerns about their suitability for large‐volume defect reconstruction. Considering these factors, the HT‐ink was developed comprising collagen (66 mg mL^−1^) supplemented with nHAp, offering biocompatibility with primary rat bone marrow stem cell (BMSCs), thermal crosslinking ability, and continuous extrudability.^[^ ^14^ ^]^ Nevertheless, the efficacy of cell incorporation through IOB remains inconclusive due to the alteration in the rheological and degradation properties of the HT‐ink. To overcome these limitations, in the current study, we advanced the HT‐ink by augmenting the collagen content to mitigate the loss of mechanical properties and improve the physical stability (Figure S1A, Supporting Information). With stable extrudability, improved storage modulus, and high biocompatibility established, we conducted a comprehensive analysis on the *in* *vitro* and in vivo effects of cell bioprinting, focusing on osteogenic activities. To induce osteogenesis, hADSCs were used for multiple reasons. hADSCs are widely selected for preclinical studies owing to their abundance and easy harvesting.^[^ ^38^ ^]^ Moreover, hADSCs have shown the capacity to differentiate toward osteogenic lineage when combined with collagen and nHAp.^[^ ^31^, ^32^ ^]^ Based on these factors, hADSCs are highly advantageous for bone tissue reconstitution.

In vitro studies have demonstrated the feasibility of the formulated HC‐ink for EBB and bone regeneration. Stable extrudability was achieved with a precise regulation of both the HT‐ and HC‐inks, even with varying collagen concentrations from 40 mg mL^−1^ in the dispersed state to 66 mg mL^−1^ in the dissolved state. When hADSCs were added, both bioinks maintained high cell viability over 90% but differed in osteogenic activities. Specifically, HC‐10 M resulted in higher Col1 and BSP expression at Day 28 via qRT‐PCR (Figure 2C,F), indicating its potential to accelerate osteogenesis when applied in vivo. In contrast, HT‐1 M and ‐5 M resulted in downregulated osteogenic gene expressions compared to those of the HC‐ink (Figure 2C–F). With this premise, we proceeded to in vivo studies to the HC‐ink.

The rationale for using different bioink compositions in two different animal models is grounded in both scientific and translational considerations. In the rat model, we incorporated hADSCs into the bioink to demonstrate the feasibility and regenerative potential of IOB. This small animal model allowed us to evaluate the biological performance of the cell‐laden construct and establish a critical foundational proof of concept for the future use of stem cells in engineering composite tissues, such as vascularized or soft–hard interface constructs. In contrast, for the large animal (sheep) model, we intentionally excluded cells from the bioink to avoid potential xenogeneic immune responses. Such immune reactions could introduce confounding variables, making it difficult to isolate and assess the performance of the bioink material itself. This model was designed to assess the scalability, surgical handling, and structural fidelity of bioprinted constructs in a clinically relevant setting. By focusing on acellular printing, we aimed to highlight the translational potential of the technology for generating patient‐specific, centimeter‐scale constructs with complex geometries.

To mitigate immune responses, T cell‐deficient RNU athymic rats were selected; these animals have previously been shown to promote bone healing when injected with human BMSC‐laden hydrogels, exhibiting nonlymphocyte morphology in contrast to immunocompetent Sprague Dawley rats.^[^ ^39^, ^40^ ^]^ Among various rodent skeletons available for evaluating bone regeneration strategies, rat calvaria serves as a well‐established, reproducible, and versatile option. While it experiences minimal biomechanical loading, its consistent anatomy and ease of surgical access make it ideal for systematically assessing bioink performance without the confounding variables introduced by fixation hardware or complex mechanical environments.^[^ ^41^ ^]^ This makes the site ideal for evaluating the efficacy of bioinks and offers a stable platform for developing complex tissues, like vascularized bone or soft–HT composite.^[^ ^14^ ^]^ Although more geometrically complex models such as mandibular defects are indeed valuable, the calvarial model serves as a critical first step in validating the bioink formulation, printability, and biological performance in vivo, even on a large animal model. The insights gained here lay the groundwork for future studies in more mechanically and anatomically demanding models. Additionally, while a single 8 mm defect per rat has been widely used in the literature, two 5 mm defects per rat also serve as a critical defect size not permitting spontaneous healing as described in many studies.^[^ ^42^, ^43^, ^44^, ^45^, ^46^ ^]^

In this work, animal studies were conducted on rats and sheep, with rats being among the most commonly used models for CMF reconstruction and sheep being the least commonly used, according to over 600 studies.^[^ ^47^ ^]^ However, when IOB via an automated platform is considered, the reported cases become rare, due to challenges of ensuring biocompatibility, printability, time efficiency, and a convenient setup suitable for surgical settings.^[^ ^11^, ^48^, ^49^ ^]^ Reported sheep calvarial defects have been created in various dimensions and shapes but with comparable volumes. For example, a square defect (35 × 35 × 3 mm),^[^ ^50^ ^]^ two square defects (20 × 20 × 5 mm),^[^ ^51^ ^]^ and four cylindrical defects (10 mm dia. × 20 mm thickness)^[^ ^52^ ^]^ had total volumes of 4000, 3675, and 3142 mm^3^, respectively, which are similar to the four counterbored‐shaped defects (4835 mm^3^) created in this study. Furthermore, applying IOB in large animal models poses challenges of intricate shapes, complex procedures, and the need for large volume of biomaterials.^[^ ^53^, ^54^ ^]^ Hence, this study is one of the earliest attempts to incorporate and thoroughly analyze IOB in both small and large animal models. Specifically, the method integrates the bioink formulation and optimization of IOB using the 3‐axis bioprinter for rats and the 6‐axis robotic arm for sheep. While the 3‐axis bioprinter is effective, it often lacks the degrees of freedom (flexibility) required for precise 3D development on nonplanar surfaces. In contrast, the 6‐axis robotic arm was chosen for its ability to streamline the procedure, generate complex‐shaped constructs, and facilitate real‐time customization, as well as its capability to facilitate large volume on large subjects.

Multiple studies have explored CMF reconstruction via IOB. For example, different bioprinting approaches have been employed using a laser‐based bioprinter, 3‐axis bioprinter (EBB), or a robotic arm (EBB) to treat rat cranial defects,^[^ ^10^, ^14^, ^37^ ^]^ achieving BV/TV of ≈13% at Week 8, ≈26% at Week 6, and ≈30% at Week 6, respectively. Our study, in comparison, achieved a BV/TV ratio of ≈36% at Week 8, notably with a ≈90% bone coverage area and significant mechanical integration. Also, among studies utilizing IOB via various bioprinting modalities, they primarily provide BV/TV values, which limit comparisons across various aspects. For sheep calvarial defects, injection^[^ ^55^ ^]^ and implantation^[^ ^56^ ^]^ were reported. This highlights the need of comprehensive investigations on IOB including thorough postoperative assessments, such as μCT imaging, mechanical testing, and histological examination, as presented in this study.

Moreover, the influence of bone regeneration potency at different cell densities was demonstrated in rats. Specifically, the HC‐ink showed improved efficacy in bone regeneration with increasing cell density up to 10 M mL^−1^. In brief, the HC‐10 M group achieved bone coverage area of ≈90% at Week 8. Subsequently, HC‐rhBMP‐2 was assessed in the small animal model to compare its efficacy to hADSC‐laden HC‐inks. Briefly, HC‐rhBMP‐2 showed comparable outcomes to the HC‐only group but was less effective than hADSC‐laden HC‐ink groups regarding BMD, bone bridging, and RUNX2 expression (Figure 3D,F, and 4C). This can be further elaborated with the literature. When poly(lactic*‐*co‐glycolic acid) (PLLA) constructs coated with BMP‐2, phenamil (a molecule activating BMP signaling pathway), or BMP‐2+phenamil were tested using mouse ADSCs, the combination of BMP‐2+phenamil induced invitro osteogenic differentiation.^[^ ^57^ ^]^ After 6 weeks of implantation, the phenamil+BMP‐2 combination markedly enhanced mouse calvarial regeneration. Notably, a reduced BMP‐2 dosage did not compromise the healing outcome,^[^ ^57^ ^]^ suggesting that BMP‐2 may function more effectively as an osteoinductive cue or transfection enhancer when combined with other agents or cells. However, rhBMP‐2 was reported to facilitate bone regeneration,^[^ ^58^ ^]^ as evidenced by increased new bone formation and enhanced RUNX2 intensity in a rat model,^[^ ^14^ ^]^ as well as improved new bone formation in a sheep model.^[^ ^58^ ^]^ In contrast, increasing hADSC density promoted rat calvarial regeneration in this study, indicating that the use of cells not only serves as a delivery vehicle but also plays a central role in facilitating bone regeneration. For the sheep model, the robotic arm assisted with pneumatic pressure generated two cylindrical compartments within ≈5 min per defect. Although cells were excluded to prevent an immune response from hADSCs, HC‐rhBMP‐2 showed promising potential for cranial reconstruction through IOB. This was evidenced by the highest bone coverage area of ≈80%. These results imply that IOB into a large bone defect is feasible, and the combination of native hADSCs and rhBMP‐2 may synergistically promote bone regeneration.

Regarding the practicality of IOB, while we acknowledge that the setup process for IOB is more complex and time‐consuming compared to manual loading of biomaterials, IOB offers critical advantages that justify its complexity—particularly in the context of precision, spatial control, and reproducibility. IOB enables automated, layer‐by‐layer deposition of bioinks directly into the defect site, allowing for the fabrication of patient‐specific constructs with defined architecture, which is difficult to achieve with manual techniques. These benefits are especially relevant for future applications involving multimaterial or cell‐laden constructs, where spatial fidelity is essential. Manual loading of biologics may serve as a counterpart in terms of preparation and operation time; for instance, the Bio‐Oss pen has yielded promising results in rat models. However, it is often associated with drawbacks such as inconsistent placement and a higher risk of ectopic bone regeneration due to biomaterial leakage.^[^ ^26^ ^]^ Also, combining biologics with cells becomes more challenging when manual intervention is involved, as it may compromise homogeneous cell distribution and cell viability. In contrast, the automation of surgical procedures—particularly involving bioprinters—can alleviate these challenges along with the usage of hADSCs mitigating prolonged preparation times by easy accessibility, high proliferative capacity, resistance to senescence, and sustained differentiation potential for a long period.^[^ ^59^ ^]^

After fabrication, evaluation of the mechanical properties of bioprinted constructs was essential, as they are critical indicators of tissue functionality. Native sheep cranial bone possesses an elastic modulus of 100–3000 MPa for cancellous bone and 10–20 GPa for compact bone.^[^ ^27^, ^60^ ^]^ Given the low load‐bearing nature of cranial bone,^[^ ^61^ ^]^ collagen can be a feasible material to induce bone regeneration and the infiltration of host tissues. Specifically, Col1, which constitutes 90% of the organic matrix, plays a crucial role in forming the hierarchical bone structures that influence its mechanical properties.^[^ ^62^ ^]^ We performed mechanical testing with the bioprinted substitutes with adjacent sheep calvarial bone (Figure S9B, Supporting Information), which resulted in 101–136 MPa Young's modulus. With an elastic modulus aligning with that of cancellous bone, the properties of the HC‐ink allowed host tissues to remodel the entire defect area and facilitated its integration with the bioprinted constructs, as evidenced by improved Young's modulus, peak force, and energy (Figure 5K–M). We posit that the collagen‐based bioink could be carefully modulated for future endeavors to facilitate bone maturation and mimic the mechanical intricacies of bone layers.

Overall, several limitations need to be addressed before proceeding to clinical translation. First, while new bone formation was observed in 8 weeks for a rat model and 3 months for a sheep model, extending the observation period could be beneficial for comparison with conventional techniques, which have a mean cranioplasty interval of 8.6 months.^[^ ^49^ ^]^ Also, effective vascularization incorporated with IOB should be devised. As defect volume increases, rapid vascularization becomes essential as it transports nutrients and wastes necessary for tissue survival and the prevention from necrosis. To date, vascularization has been attempted via IOB using endothelial cells;^[^ ^10^ ^]^ however, successful anastomosis with host vasculature and achieving functional integration remain significant challenges for bone regeneration. In this regard, neovascularization can be induced by combining IOB with surgical techniques, such as arteriovenous loop, vascular pedicles, or micropuncture (MP). Of these, MP demonstrated promising results, achieving rapid vascularization across bulk collagen implants within 24 h by creating MPs in the host vasculature of rat.^[^ ^63^ ^]^ It aligns well with the goal of IOB, which focuses on rapid tissue repair and restoration of tissue function. Concurrently, future studies should aim to provide precise guidance for cell differentiation and streamlined procedures to create complex composite scalable tissues like vascularized and/or reinnervated bone.

From a clinical standpoint, although not yet universally available, IOB holds immense potential to address the existing challenges associated with conventional CMF surgical practices. For example, several clinical cases for human CMF defect reconstruction were reported using nonbiological materials (i.e., polymethylmethacrylate (PMMA), titanium, and hydroxyapatite).^[^ ^64^ ^]^ While these implants offer ease of fabrication and biocompatibility, certain drawbacks—such as brittle nature of PMMA, nonabsorbable properties of titanium, or limitations in cellular remodeling—can lead to serious complications, including infection or damage to the delicate brain tissue beneath. Moreover, real‐time intraoperative modification becomes critical in cases where unexpected discrepancy may arise, such as tumor removal. In this regard, IOB offers rapid surgical procedures and enables real‐time adjustments to implants. Moreover, those nonbiological materials are limited to reconstitute adjacent soft tissues, such as skin and periosteum. After placing implants in the defect area, there remains a risk of a sentinel bleed, especially due to a brain aneurysm, unless the defect site is securely covered and stabilized with soft tissues. As IOB is not limited to synthetic or biological materials, it presents a promising alternative for mimicking and reconstructing various tissue types in situ. Given the encouraging results in CMF reconstruction in a large animal model, advancements in IOB, along with addressing current challenges such as vascularized hard/soft composite tissues, will be the next milestone toward realizing clinical translation.

## 4 Conclusion

In conclusion, we have successfully implemented the IOB technology for CMF repair, progressing from a rat model to a sheep model. The bioink specific to HT was formulated with a high collagen content, supporting bioprintability by including cells and a safe crosslinking process. The implantation of IOB and the bioink facilitated new bone formation and mechanical enhancement. Taken together, our findings suggest the potential for the effective reconstruction of large calvarial bone defects, marking a significant technical leap toward precisely developed 3D implants using advanced bioprinted platforms for surgical intervention.

## 5 Experimental Section

5.1

5.1.1

##### Study Design

This study was designed to investigate IOB for calvarial bone regeneration and its applications in a small and large animal model. We used our previously established HT‐ink and also formulated the HC‐ink to facilitate the incorporation of hADSCs without compromising the rheological and bioprinting capabilities. To evaluate the feasibility of IOB of bioinks, a thorough in‐vitro investigation was conducted first, including cell viability, morphology, and osteogenic activities via immunofluorescent imaging and qRT‐PCR. After observing cell survival and osteogenesis for 4 weeks of in‐vitro cultivation, in vivo tests were carried out using immunocompromised rats. Two critical‐size calvarial bone defects (each with a dia. of 5 mm) were created in rats. Then, a total of eight groups was assessed using eight defects (*n* = 8) at different bioprinting conditions, including control (empty), HT‐ink (without cells, with 1, and 5 million hADSCs mL^−1^) groups, HC‐ink (without cells, with rhBMP‐2, and 1, 5, and 10 million hADSCs mL^−1^), and Bio‐Oss. At Week 8, characterizations using μCT scanning, histomorphometric analysis, and a compression test of bone specimens displayed different bone healing progress. Furthermore, on sheep calvaria, four counterbored defects were formed in two cylindrical compartments with diameters of 14 and 11 mm. Three groups were established, namely control (empty) (*n* = 8), HC‐ink (*n* = 8), and HC‐ink + BMP‐2 (*n* = 8), and each group was allocated a male and a female sheep for the study. Assessment of the sheep study was performed at Week 12. All in vitro and in vivo results were replicated at least three times unless otherwise stated.

##### Statistical Analysis

Data were represented using GraphPad Prism 8 (GraphPad Software, Inc.) software and statistically analyzed using SPSS 29 (IBM) software. The data were presented in mean ± standard deviation unless otherwise stated. Multiple group comparison was performed by one‐way analysis of variance (ANOVA) with posthoc Tukey tests. Statistical differences were considered significant at *p < 0.05, **p < 0.01, and ***p < 0.001. Comparison between two groups was performed by independent‐samples T test, where statistical differences were considered at ^#^ *p* < 0.05, ^##^ *p* < 0.01, ^###^ *p* < 0.001 or ^‡^ *p* < 0.05, ^‡‡^ *p* < 0.01, ^‡‡‡^ *p* < 0.001. Comparison between two time points within the same group was performed by paired‐samples T test, where statistical differences were considered at ^†^ *p* < 0.05, ^††^ *p* < 0.01, and ^†††^ *p* < 0.001.

## Conflict of Interest

I.T.O. serves as a scientific advisor for Biolife4D and Healshape and owns stock in Biolife4D. All other authors declare no competing interests.

## Author Contributions

**Miji Yeo**: data curation (lead); formal analysis (lead); investigation (lead); methodology (lead); validation (lead); visualization (lead); writing—original draft (lead); writing—review and editing (lead). **Deepak Gupta**: data curation (equal); investigation (equal); methodology (equal); validation (equal); writing—original draft (equal); writing—review & editing (equal). **Irem Deniz Derman**: investigation (supporting); methodology (supporting) sendegul yildirim (investigation (supporting); methodology (supporting). **Yogendra P. Singh**: investigation (supporting); methodology (supporting); writing—review & editing (supporting). **Ethan Michael Gerhard**: investigation (supporting); methodology (supporting). **Elias Rizk**: investigation (supporting); methodology (supporting). **Thomas Neuberger**: (investigation (supporting); methodology (supporting); visualization (supporting). **Scott Simon**: investigation (supporting). **Ibrahim T. Ozbolat**: conceptualization (lead); funding acquisition (lead); investigation (lead); methodology (lead); project administration (lead); supervision (lead); validation (lead); writing—review and editing (lead). **Miji Yeo** and **Deepak Gupta** contributed equally to this work.

## Supporting information

Supplementary Material

## Data Availability

The data that support the findings of this study are available in the article and its Supporting Information.

## References

[1] A. Marchant , S. Allyn , A. Burke , A. Gaal , J. Dillon , J. Oral Maxillofacial Surg. 2024, 82, 199. 10.1016/j.joms.2023.11.011 38040026

[2] S. N. Khan , F. P. Cammisa Jr , H. S. Sandhu , A. D. Diwan , F. P. Girardi , J. M. Lane , J. Am. Acad. Orthop. Surg. 2005, 13, 77. 15712985

[3] G. Liu , Y. Zhang , B. Liu , J. Sun , W. Li , L. Cui , Biomaterials 2013, 34, 2655. 23343633 10.1016/j.biomaterials.2013.01.004

[4] J. Zheng , Z. Zhao , Y. Yang , S. Wang , Y. Zhao , Y. Xiong , S. Yang , Z. Qui , T. Song , C. Zhang , Regener. Biomater. 2022, 9, rbac004. 10.1093/rb/rbac004 PMC9113234 35592140

[5] U. Hubbe , S. Beiser , S. Kuhn , Biomater. Adv. 2022, 136, 212754. 35929289 10.1016/j.bioadv.2022.212754

[6] J.‐R. Kuo , C.‐C. Wang , C.‐C. Chio , T.‐J. Cheng , J.Clin. Neurosci. 2004, 11, 486. 15177389 10.1016/j.jocn.2003.06.005

[7] D. J. Prolo , J. J. Rodrigo , Clin. Orthop. Relat. Res. 1976‐2007 1985, 200, 322. 3905118

[8] C.‐H. Hou , R.‐S. Yang , S.‐M. Hou , J. Hosp. Infect. 2005, 59, 41. 15571852 10.1016/j.jhin.2004.03.017

[9] S. V. Murphy , A. Atala , Nat. Biotechnol. 2014, 32, 773. 25093879 10.1038/nbt.2958

[10] O. Kérourédan , D. Hakobyan , M. Rémy , S. Ziane , N. Dussere , J.‐C. Fricain , S. Delmond , N. B. Thébaud , R. Devillard , Biofabrication 2019, 11, 045002. 31151125 10.1088/1758-5090/ab2620

[11] L. Li , J. Shi , K. Ma , J. Jin , P. Wang , H. Liang , Y. Cao , X. Wang , Q. Jiang , J. Adv. Res. 2021, 30, 75. 34026288 10.1016/j.jare.2020.11.011 PMC8132211

[12] G. Seifring Jr , A. Apostol , Biochemistry 1978, 17, 2598. 28146 10.1021/bi00606a022

[13] S. Raees , F. Ullah , F. Javed , H. M. Akil , M. J. Khan , M. Safdar , I. U. Din , M. A. Alotaibi , A. I. Alharthi , M. A. Bakht , A. Ahmad , A. A. Nassar , Int. J. Biol. Macromol. 2023, 232, 123476. 36731696 10.1016/j.ijbiomac.2023.123476

[14] K. K. Moncal , H. Gudapati , K. P. Godzik , D. N. Heo , Y. Kang , E. Rizk , D. J. Ravnic , H. Wee , D. F. pepley , V. Ozbolat , G. S. Lewis , J. Z. Moore , R. R. Driskell , T. D. Samson , I. T. Ozbolat , Adv. Funct. Mater. 2021, 31, 2010858. 34421475 10.1002/adfm.202010858 PMC8376234

[15] K. K. Moncal , M. Yeo , N. Celik , T. M. Acri , E. Rizk , H. Wee , G. S. Lewis , A. K. Salem , I. T. Ozbolat , Biofabrication 2022, 15, 015011. 10.1088/1758-5090/ac9f70 PMC10012389 36322966

[16] M. Bitar , R. A. Brown , V. Salih , A. G. Kidane , J. C. Knowles , S. N. Nazhat , Biomacromolecules 2008, 9, 129. 18095652 10.1021/bm701112w

[17] B. de Campos Vidal , M. L. S. Mello , Micron 2011, 42, 283. 21134761 10.1016/j.micron.2010.09.010

[18] A. Chandrasekar , S. Sagadevan , A. S. Dakshnamoorthy , Int. J. Phys. Sci. 2013, 8, 1639.

[19] T. Komori , in Osteoimmunology: Interactions of the Immune and Skeletal Systems II 2010, Springer, New York, p. 43.

[20] L. Malaval , N. M. Wade‐Guéye , M. Boudiffa , J. Fei , R. Zirngibl , F. Chen , N. Laroche , J.‐P. Roux , B. Burt‐Pichat , F. Duboeuf , G. Boivin , P. Jurdic , M.‐H. Lafage‐Proust , J. Amédée , L. Vico , J. Rossant , J. E. Aubin , J. Exp. Med. 2008, 205, 1145. 18458111 10.1084/jem.20071294 PMC2373846

[21] L. Tong , Q. Liao , Y. Zhao , H. Huang , A. Gao , W. Zhang , X. Gao , W. Wei , M. Guan , P. K. Chu , H. Wang , Biomaterials 2019, 193, 1. 30550998 10.1016/j.biomaterials.2018.12.008

[22] D. G. Monroe , J. R. Hawse , M. Subramaniam , T. C. Spelsberg , BMC Musculoskeletal Disord. 2010, 11, 1. 10.1186/1471-2474-11-104 PMC2888765 20509905

[23] Z. S. Patel , S. Young , Y. Tabata , J. A. Jansen , M. E. Wong , A. G. Mikos , Bone 2008, 43, 931. 18675385 10.1016/j.bone.2008.06.019 PMC3014108

[24] Y. Kang , M. Yeo , I. D. Derman , D. J. Ravnic , Y. P. Singh , M. A. Alioglu , Y. Wu , J. makkar , R. R. Driskell , I. T. Ozbolat , Bioact. Mater. 2024, 33, 114. 38024230 10.1016/j.bioactmat.2023.10.034 PMC10665670

[25] A. Pliss , L. Zhao , T. Y. Ohulchanskyy , J. Qu , P. N. Prasad , ACS Chem. Biol. 2012, 7, 1385. 22594453 10.1021/cb300065w

[26] B.‐H. Yu , Q. Zhou , Z.‐L. Wang , J. Biomater. Appl. 2014, 29, 243. 24487130 10.1177/0885328214521846

[27] S. Wang , Y. Yang , Z. Zhao , X. Wang , A. G. Mikos , Z. Qiu , T. Song , X. Sun , L. Zhao , C. Zhang , ACS Biomater. Sci. Eng. 2017, 3, 1092. 33429583 10.1021/acsbiomaterials.7b00159

[28] F. L. Garcia , C. H. Picado , S. B. Garcia , Arch. Orthop. Trauma Surg. 2009, 129, 549. 18297297 10.1007/s00402-008-0587-9

[29] M.‐E. Marquis , E. Lord , E. Bergeron , O. Drevelle , H. Park , F. cabana , H. Senta , N. Faucheux , Front. Biosci. 2009, 14, 1023. 10.2741/3293 19273115

[30] T. Komori , Int. J. Mol. Sci. 2019, 20, 1694.30987410

[31] Y. Li , Y. Liu , R. Li , H. Bai , Z. Zhu , L. Zhu , C. Zhu , Z. Che , H. Liu , J. Wang , L. Huang , Mater. Des. 2021, 210, 110049.

[32] G. Calabrese , R. Giuffrida , C. Fabbi , E. Figallo , D. L. Furno , R. Gulino , C. Colarossi , F. Fullone , R. Giuffrida , R. Parenti , L. Memeo , S. Forte , PLoS one 2016, 11, e0151181. 26982592 10.1371/journal.pone.0151181 PMC4794180

[33] M.‐K. Hsieh , C.‐J. Wu , C.‐C. Chen , T.‐T. Tsai , C.‐C. Niu , S.‐C. Wu , P.‐L. Lai , Mater. Sci. Eng.: C 2018, 91, 806. 10.1016/j.msec.2018.06.004 30033316

[34] C. Zhang , B. Yan , Z. Cui , S. Cui , T. Zhang , X. Wang , D. Liu , R. Yang , N. Jiang , Y. Zhou , Sci. Rep. 2017, 7, 10519. 28874877 10.1038/s41598-017-11155-7 PMC5585269

[35] Anatomy and Ultrastructure of Bone – Histogenesis, Growth and Remodeling, (Massachusetts: MDText.com, Inc., South Dartmouth , 2015. 25905372

[36] B. Xu , P. Zheng , F. Gao , W. Wang , H. Zhang , X. Zhang , X. Feng , W. Liu , Adv. Funct. Mater. 2017, 27, 1604327.

[37] M. Xie , Y. Shi , C. Zhang , M. Ge , J. Zhang , Z. Chen , J. Fu , Z. Xie , Y. He , Nat. Commun. 2022, 13, 3597. 35739106 10.1038/s41467-022-30997-y PMC9225998

[38] A. N. Rindone , B. Kachniarz , C. C. Achebe , R. C. Riddle , A. N. O'Sullivan , A. H. Dorafshar , W. L. Grayson , Adv. Healthcare Mater. 2019, 8, 1801565. 10.1002/adhm.201801565 30941920

[39] L. P. Hatt , A. R. Armiento , K. Mys , K. Thompson , M. Hildebrand , D. Nehrbass , W. E. G. Müller , S. Zeiter , D. Eglin , M. J. Stoddart , Acta Biomaterialia 2023, 156, 177. 35988660 10.1016/j.actbio.2022.08.021

[40] D. S. Garske , K. Schmidt‐Bleek , A. Ellinghaus , A. Dienelt , L. Gu , D. J. Mooney , G. N. Duda , A. Cipitria , Tissue Eng. Part A 2020, 26, 852. 32046626 10.1089/ten.TEA.2019.0310

[41] P. P. Spicer , J. D. Kretlow , S. Young , J. A. Jansen , F. K. Kasper , A. G. Mikos , Nat. Protoc. 2012, 7, 1918. 23018195 10.1038/nprot.2012.113 PMC3513397

[42] P. Gomes , M. Fernandes , Lab. Anim. 2011, 45, 14. 21156759 10.1258/la.2010.010085

[43] M. Pei , J. Li , D. McConda , B. S. Wen , N. B. Clovis , S. S. Danley , Bone 2015, 78, 1. 25940459 10.1016/j.bone.2015.04.040 PMC4466199

[44] S. J. Florczyk , M. Leung , Z. Li , J. I. Huang , R. A. Hopper , M. Zhang , J. Biomed. Mater. Res. Part A 2013, 101, 2974. 10.1002/jbm.a.34593 23737120

[45] H. Honda , N. Tamai , N. Naka , H. Yoshikawa , A. Myoui , J. Artif. Organs 2013, 16, 305. 23700004 10.1007/s10047-013-0711-7

[46] J.‐H. Kim , H.‐W. Kim , Tissue Eng. Regener. Med. 2013, 10, 310.

[47] M. Maroulakos , G. Kamperos , L. Tayebi , D. Halazonetis , Y. Ren , J. Dent. 2019, 80, 1. 30439546 10.1016/j.jdent.2018.11.004

[48] W. Zhao , C. Hu , T. Xu , Bioact. Mater. 2023, 25, 201. 36817820 10.1016/j.bioactmat.2023.01.018 PMC9932583

[49] C. Unterhofer , C. Wipplinger , M. Verius , W. Recheis , C. Thomé , M. Ortler , Neurol. Neurochir. Pol. 2017, 51, 214. 28343651 10.1016/j.pjnns.2017.02.007

[50] P. A. Gerety , J. D. Wink , R. D. Sherif , N. Clarke , H.‐D. Nah , J. A. Taylor , J. Craniofacial Surg. 2014, 25, 1917. 10.1097/SCS.0000000000000987 25119411

[51] J. O. Voss , S. Kasselmann , S. Koerdt , C. Rendenbach , H. Fischer , K. Joehrens , M. Czabanka , K. Schmidt‐Bleek , G. Duda , M. N. Heiland , Biomater. Adv. 2022, 136, 212788. 35929320 10.1016/j.bioadv.2022.212788

[52] C. Adamzyk , P. Kachel , M. Hoss , F. Gremse , A. Modabber , F. Hölzle , R. Tolba , S. Neuss , B. Lethaus , J. Cranio‐Maxillofacial Surg. 2016, 44, 985. 10.1016/j.jcms.2016.04.012 27328894

[53] A. De Pieri , Byerley A. M., C. R. Musumeci , F. Salemizadehparizi , M. A. Vanderhorst , K. Wuertz‐Kozak , JOR Spine 2020, 3, e1117. 33392454 10.1002/jsp2.1117 PMC7770193

[54] A. G. Tabriz , M. A. Hermida , N. R. Leslie , W. Shu , Biofabrication 2015, 7, 045012. 26689257 10.1088/1758-5090/7/4/045012

[55] J. M. Townsend , E. A. Kiyotake , J. T. Easley , H. B. Seim III , H. L. Stewart , K.‐M. Fung , M. S. Detamore , Materialia 2023, 27, 101690. 36743831 10.1016/j.mtla.2023.101690 PMC9897238

[56] S. Wang , Z. Zhao , Y. Yang , A. G. Mikos , Z. Qiu , T. Song , F. Cui , X. Wang , C. Zhang , Regener. Biomater. 2018, 5, 283. 10.1093/rb/rby020 PMC6184757 30338126

[57] J. Fan , C. S. Im , Z.‐K. Cui , M. Guo , O. Bezouglaia , A. Fartash , J.‐Y. Lee , J. Nguyen , B. M. Wu , T. Aghaloo , M. Lee , Tissue Eng. Part A 2015, 21, 2053. 25869476 10.1089/ten.tea.2014.0489 PMC4507130

[58] A. M. Boos , A. Weigand , G. Deschler , T. Gerber , A. Arkudas , U. Kneser , R. E. Horch , J. P. Beier , Int. J. Nanomed. 2014, 9, 5317, 10.2147/IJN.S66867. PMC4242408 25429218

[59] Q. Le , V. Madhu , J. M. Hart , C. R. Farber , E. R. Zunder , A. S. Dighe , Q. Cui , World J. Stem Cells 2021, 13, 1248. 34630861 10.4252/wjsc.v13.i9.1248 PMC8474721

[60] PEEK Biomaterials Handbook, 2nd ed., William Andrew, New York 2019.

[61] S. Boruah , K. Henderson , D. Subit , R. S. Salzar , B. S. Shender , G. Paskoff , in Proc. of the IRCOBI Conf. 2013, p. 497.

[62] P. Garnero , Calcif. Tissue Int. 2015, 97, 229. 25894071 10.1007/s00223-015-9996-2

[63] P. C. Hancock , S. V. Koduru , M. Sun , D. J. Ravnic , Microvasc. Res. 2021, 134, 104121. 33309646 10.1016/j.mvr.2020.104121

[64] J. Brie , T. Chartier , C. Chaput , C. Delage , B. Pradeau , F. Caire , M.‐P. Boncoeur , J.‐J. Moreau , J. Cranio‐Maxillofacial Surg. 2013, 41, 403. 10.1016/j.jcms.2012.11.005 23218977

