# Supplementary material for: Intraoperative Bioprinting for Craniomaxillofacial Bone Reconstruction in Rats and Sheep
Source: Small Sci. 2025 Sep 9;5(11):2400621. doi: 10.1002/smsc.202400621 (PMC12622550; doi:10.1002/smsc.202400621)
Supplement: Supplementary file 1 — Supplementary Material [file SMSC-5-2400621-s001.zip › smsc202400621-sup-0001-SuppData-S1.pdf]

Supplementary Materials for

Intraoperative Bioprinting for Craniomaxillofacial Bone Reconstruction in Rats and Sheep

Miji Yeo *et al.*

The PDF file includes:

Materials and Methods

Figs. S1-S11

Tables S1 to S4

Movies S1 to S3

References (1-11)

Materials & Methods

Materials

Stocked chitosan (CS) in powder form was purchased from Sigma-Aldrich (cat. no. 417963h) and sterilized using gamma-ray irradiation (25 kGy ± 10%, Cobalt-60, Gamma Irradiation Facility, Radiation Science and Engineering Center, The Pennsylvania State University (PSU)).[1] After evaporating a 15 w/v% aqueous hydroxyapatite solution (nanoXIM-Hap202, Fluidinova) to obtain nano-hydroxyapatite powder (nHAp), the powder was sifted using 106 μm filters and sterilized using gamma-ray irradiation, as previously described.[2] β-Glycerophosphate disodium salt hydrate (β-GP, Sigma-Aldrich) was dissolved in 1× Dulbecco's phosphate-buffered saline (DPBS, Cellgro) at 56 w/v% and sterilized using 0.22 μm filters (Millipore). Collagen type-I (ColI) was prepared by extracting Col1 from rat tail tendon as described,[3] which was then dissolved in 0.02N acetic acid (Sigma-Aldrich) at 9 mg/mL and sterilized via dialysis,[4] followed by storing at 4 °C for further use. For collagen sponges, ColI was frozen at -80 °C overnight and lyophilized (Labconco FreeZone 18 L System, Labconco) for 2-3 days. Subsequently, collagen sponges were ground using a Krups F203 grinder, and 40 mg stock packages were prepared and sterilized using an Anprolene gas sterilizer (Anderson Sterilizers, cat. no. AN74ix) supplied with ethylene oxide[5] and stored at -80 °C. Carrier free (bovine serum albumin (BSA)-free) recombinant human bone morphogenetic protein-2 (rhBMP-2) (cat. no. 355-BM-100/CF, R&D Systems) was aliquoted in sterile hydrochloric acid (4 mM, HCL, Millipore) at 30 μg/150 μL and stored at -30 °C. Bio-Oss pens (20116; 0.25-1 mm granule size; 0.5 cc) were purchased from Geistlich (Switzerland) and were added with 1 mL sterile saline according to the manufacturer's instructions before use. Carrier-free (bovine serum albumin-free) recombinant human bone morphogenetic protein-2

(rhBMP-2) was purchased from Fisher Scientific (cat. no. 355BM050CF), aliquoted at 5 µg in 25 µL of sterile 4 mM hydrochloric acid (HCl; Sigma-Aldrich), and stored at -30 °C.

Preparation of the hard-tissue ink (HT-ink) and highly-concentrated collagen ink (HC-ink)

For the HT-ink, the prepared materials were formulated according to our previous study.[6] Briefly, 200 mg CS powder was dissolved in 9 mL HCL (0.1N) and neutralized by adding 1 mL β-GP (56 w/v%) on a magnetic stirrer overnight in sterile conditions at room temperature (RT) (**Figure S1A**). Then, 1.2 mL CS-β-GP solution was homogeneously mixed with 0.3 mL sterile collagen solution (9 mg/mL). Sterile 40 mg collagen sponges and 160 mg nHAp were added to the mixture and dispersed using an in-house built mixer for 45 min. These conditions were established and verified to satisfy bioprintability and mechanical stability.[6]

For the HC-ink, consistently high-concentrated collagen solution was prepared by dissolving 100 mg/mL collagen in 0.1 N HCL in a dual syringe assembly connected with a three-way stopcock for a day (**Figure S1A**). Subsequently, the collagen was manually mixed by performing alternate injections through the three-way stopcock until homogeneity was achieved, which took approximately 2-3 days. To prevent overheating, the number of syringe pushes did not exceed 200 per round. The entire process was conducted in a cold room (4 °C). Next, 0.5 mL CS-β-GP solution was added to 1 mL sterile collagen solution in a biosafety cabinet before experiments. A total of 100 syringe pushes were performed at 4 °C. In the biosafety cabinet, 160 mg nHAp was added to form the final HC-ink after 50 syringe pushes. The final collagen concentration attained was 40 mg/mL in dispersed form for the HT-ink and 66.6 mg/mL in dissolved form for the HC-ink (**Figure S1A**). To prepare the rhBMP-2-bioink, 37.5 µg rhBMP-2 was added into 1.5 mL of HC-ink achieve a loading concentration of 1 µg per rat defect and 30 µg per sheep defect.

Fourier Transform Infrared Spectrophotometry (FTIR)

The components comprising the HT-ink and HC-ink were analyzed regarding functional groups using Attenuated Total Reflectance (ATR)-FTIR (Bruker Vertex V70 FTIR spectrometer, Germany). The heating rate was set at 20 °C per min under 100 cm3/min air gas flow rate. A spectral range of 500-4000 cm^{-1} was scanned with 100 points recorded per sample at a resolution of 0.48 cm^{-1}.

Printability

To evaluate printability using the HT-ink and HC-ink, constructs were printed under the conditions listed in **Table S1**. The target diameter and pore area were 500 μm and 1 mm^2, respectively, and three filaments were randomly selected, with 10 measurements per filament.

Diffusion rate (D_{fr}) and printability (P_r) were evaluated by the equation:

$$D_{f_r} = \frac{S_t - S_a}{S_t} \times 100\%$$

$$P_r = \frac{L^2}{16S_a}$$

where, S_t was the theoretical area of a pore, S_a was the actual area of a pore, and L was the perimeter of the actual pore.

Compression test on bioprinted constructs

The HT-ink and HC-ink were cast into 3D printed hollow cylindrical molds, each with 10 mm in diameter and 2.5 mm in height. After crosslinking at 37 °C for 30 min, the samples (n = 4) were compressed at 10 mm/s speed up to 60% of the height of constructs performed at 22 °C. Then, the force-strain graph was plotted.

Swelling and degradation

The initial dry weight was measured after printing HT-ink and HC-ink constructs. For swelling tests, samples (n = 6) were immersed in DPBS at 37 °C for 3 h and measured for their weights. For the degradation study, manually cast (n = 6) constructs and bioprinted constructs (n = 6) were used. Three samples of each group were immersed in DPBS or Collagenase type 1 at 1 mg/mL (125 U/mg; Millipore, US) and kept at 37 °C for 8 weeks. Before measuring weights, excess buffers were absorbed using KimWipe, and measurement conditions were kept consistent throughout the experiments. The following equations were used to obtain swelling and degradation rates:

$$Swelling\ rate\ (\%) = [\frac{W_s - W_i}{W_s}] \times 100$$

$$Degradation\ rate\ (\%) = [\frac{W_0 - W_f}{W_0}] \times 100$$

where, W_i was the initial weight, W_s was the swollen weight, W_0 was the initial weight, and W_f was the final weight.

Cell culture

Human adipose-derived stem cells (hADSCs) were purchased from Lonza (PT-5006). hADSCs were cultured using a DMEM/F12 1:1 medium with HEPES and without L-glutamine (SH30126.FS, Cytiva) supplemented with 20% fetal bovine serum (FBS) and 1% penicillin (100 IU/mL)-streptomycin (100 µg/mL) (cat. no. 30-002-CI, Corning Life Sciences). Cell culture flasks were then kept in an incubator in humidified 5% CO_2 at 37 °C for expansion and further use. 3D Bioprinted constructs were cultured using the same conditions described above.

To embed cells, the formulated HT-ink and HC-ink were stored at room temperature for 1 h and loaded with hADSCs under sterile conditions. Briefly, after trypsinization, the pellet of hADSCs was resuspended into single cells and counted. The suspension was adjusted to 7.5 million cells in 200 μL DMEM/F12 medium. The entire volume of adjusted hADSCs were then added into 1.5 mL bioink through the syringe orifice and gently mixed by 20 pushes through a 3-way stopcock.

Transduction of hADSCs

hADSCs were transduced with Tandem-dimer tomato (tdTomato; Vectalys, France) lentivirus at passage 4 with around 50% confluency. A transduction mix was prepared by mixing non-supplemented media, polybrene (Sigma, US) at 800 μg/mL, and a viral vector solution at a multiplicity of infection of 20. hADSCs were treated with the transduction mix for 8 h, which was then discarded. After rinsing with DPBS, transduced hADSCs were sorted using a MoFlo Astrios sorter (Beckman Coulter, US). The collected cells were cultured and expanded for further use.

Rheological properties of bioinks

Rheological properties of bioinks were evaluated using an MCR 302 rheometer (Anton Paar) connected with a cone plate (1° cone angle and 25 mm dia.) at 25 °C. For the following tests, HT- and HC-inks with and without ADSCs at 5M/mL were used. A rotational test was utilized to generate the viscosity curve in a shear rate range of 0.015 to 1000 s^{-1}. An oscillatory test was carried out using amplitude sweep of 0.01 to 1,000% strain at a 1-Hz constant frequency to measure the storage (G') and loss (G'') modulus. A strain amplitude sweep test was applied to get a curve involving complex viscosity (η^*), G' and G'' for a frequency ranging from 0.1 to 20 Hz at a 0.5% constant shear strain.

Extrusion-based bioprinting (EBB) of bone constructs *in vitro*

For in-vitro studies, bioprinting conditions were established and applied for the HT- and HC-inks using a 3-axis bioprinter (an in-house developed ‘Multi-arm Bioprinter’,[6] which were listed in **Table S1.**[2] These conditions were carefully adjusted to attain continuous flow and were feasible for both bioink types. Designed by a Python programming language, grid structures in dual layers were bioprinted to test the bioprintability and observe cellular activities.

LIVE/DEAD assay

Cell-embedded constructs were stained using the LIVE/DEAD assay on Days 1, 4, 7, and 14 to study the biocompatibility of bioinks. A mixture of calcein AM (0.15 mM, Thermo Fisher Scientific) and ethidium homodimer-1 (EtDH; 2 mM, Invitrogen) was prepared, and cell-laden constructs were incubated with the working solution for 30 min at 37 °C with 5% CO_2. More than five random ROIs were selected and captured from three technical replicates. The stained cells were then imaged using an Axio Zoom fluorescent microscope (Zeiss) with excitation wavelengths of 475 nm and 555 nm and exposure times of 15 ms and 10 ms for calcein and EtDH, respectively. Post-capture adjustments were applied through deconvolution to reduce background noise while ensuring all cells were retained. Cell viability (%) was calculated as the proportion of live (green) cells to the total cell count, encompassing both live and dead (red) cells.

Immunofluorescent (IF) imaging

Cell morphology and osteogenic activities were represented on Days 14 and 28 by cytoskeletal and IF imaging, respectively. Firstly, hADSCs were fixed for overnight using 4% paraformaldehyde at 4 °C. Followed by permeabilization with 0.3% Triton X-100 (Sigma-Aldrich) in Dulbecco’s Phosphate-Buffered Saline (DPBS) for 30 min, cells were treated using 10% normal goat serum (Abcam) in DPBS at RT for 2 h. Mouse anti-runt-related transcription factor 2 (RUNX2)

antibody (1:500 in DPBS, cat. no. ab76956, Abcam) and rabbit anti-bone sialoprotein (BSP) antibody (1:500 in DPBS, cat. no. ab52128, Abcam) were used to conjugate osteogenic-specific proteins overnight at 4 °C. The secondary antibodies of Alexa Fluor 488 goat anti-mouse (1:500 in DPBS, cat. no. A11017, Invitrogen) for RUNX2 and Alexa Fluor 647 goat anti-rabbit (1:500 in DPBS, cat. no. A21245, Invitrogen) for BSP were used for 2 h along with Phalloidin (cat. no. A12380, Molecular Probes) for cytoskeleton and Hoechst (Life Technologies) for nuclei. To verify background staining, acellular constructs were treated and stained using the same conditions used for cell-laden bioprinted constructs The images were captured using a Zeiss LSM880 confocal microscope (Zeiss).

Gene expression via quantitative real-time polymerase chain reaction (qRT-PCR)

The primers of collagen type 1 (Col1), RUNX2, Osterix, and BSP were utilized as listed in **Table S4.**[7] Briefly, Trizol reagent (Thermo Fisher Scientific) was used to isolate RNA from cells, in which RNA concentrations were determined using a NanoDrop (ND-1000 Spectrophotometer) at 260/280 mm. According to the AccuPower protocol, complementary deoxyribonucleic acid (cDNA) was synthesized using T100 Thermal Cycler (BioRad). Thereafter, a StepOnePlus Real-Time PCR System (Applied Biosystems) was utilized with Power SYBRTM Green Master Mix (Thermo Fisher Scientific). The prepared genes were loaded in a 96-well plate with at least triplicates, which was analyzed using the comparative cycle threshold method. Shortly, the target genes were quantified using the $2^{-\Delta\Delta CT}$ method and normalized with respect to the housekeeping gene, glyceraldehyde 3-phosphate dehydrogenase (GAPDH). hADSCs cultured in 2D were extracted on Day 1 as a control and compared with 3D bioprinted hADSCs on Day 28 to quantify the expression of osteogenic-related gene markers. The fold-change of 3D bioprinted hADSCs relative to the 2D hADSCs was represented as the mean ± standard deviation.

Surgical procedures for rats

A total of 32 inbred 11-12 weeks-old female RNU athymic (Crl:NIH-Foxn1rnu, Charles River) rats were obtained and housed in our animal facility at Millennium Science Complex (PSU) in accordance with the guidance set by American Association for Laboratory Animal Science (AALAS) and the Institutional Animal Care and Use Committee (IACUC; protocol #46591). Rats were anesthetized using 2-3% isoflurane throughout the surgical procedure. After deep anesthesia, heads were shaved and artificial tears (Rugby Laboratories) were applied to both eyes. The surgical sites were cleansed using ethanol and betadine. Prior to incision, 0.15-0.2 mL Bupivacaine ER (0.5%, ZooPharm) was injected under the skin. The details of groups, number of defects, and observation periods were listed in **Table S4**.

A sagittal incision (~2 mm) was made, which exposed the periosteum, and the skin was pulled back and fixed. The calvarium was exposed through the sagittal incision of the periosteum, which was retracted immediately. A drill with a trephine bit was utilized to create two critical-sized calvarial defects (each with 5 mm in dia.) into the parietal bone on each side of the rat skull, with careful attention to preserving the integrity of the Dura mater. The same treatment group was applied for the two defects within the same animal. The bioprinting conditions were acquired from the in-vitro studies (**Table S1**) and employed to execute a path plan in a circular pattern. An offset of 700 μm inwards of the center of the defect was given to the outline of the perimeter to avoid collision between the defect periphery and the nozzle. The final path plan was converted into the G code and transferred to the 3-axis bioprinter.

Micro-computed tomography (μCT) of rat calvarial defects

μCT scanning was performed using a μCT scanner (SkyScan 1176; Bruker Micro-CT) to evaluate the progress of bone repair. For in-vivo scanning at Week 4, the total scanning time was ~15 min per rat, and anesthesia was monitored via the breathing rate with the supply of 2-3% isoflurane. Scanning was performed using a 0.5 mm aluminum filter, 18 mm isometric voxels, 80 kV energy, 320 ms exposure, 0.5° rotation degree, and 278 mA intensity. At Week 8, euthanized rats were scanned in the same manner. After scanning, images were reconstructed using Nrecon Reconstruction software (Micro Photonics Inc.) and visualized using Avizo software (FEI Company). Specifically, a phantom with different nHA concentrations (0, 200, 400, 600, and 800 mmHA/ccm) was scanned to generate a standard curve for assessing bone mineral density (BMD). After marking the surgical sites (5-mm defect area), the regenerated bone was filtered using BMD above 300 mmHA/ccm for identifying the hard bone, which was indicated in blue. The marked region was analyzed in bone volume divided by total volume (BV/TV (%)), normalized bone mineral density (%), and bone coverage area (%). Subsequently, the bony bridging was evaluated as an index to score regenerated bone morphology from 0 (no bone formation) to 4 (bony bridging of entire span at longest point (5 mm)) according to a previous study.[8]

Histological analysis of rat samples

Bone explants at Week 8 were separated from the surrounding soft tissue for subsequent histological analysis. After rinsing with DPBS, explants were fixed with 4% paraformaldehyde for 2 days and decalcified using 0.5 M ethylenediaminetetraacetic acid (EDTA) disodium salt (Research Products International) solution for 6 weeks. The samples were then encapsulated in an O.C.T cryomatrix (Thermo Fisher Scientific) embedding resin for sectioning via a Leica CM1950 cryostat (Leica Biosystems) with 18 μm thickness at -25 °C. An automated process without heat was carried out for Haemotoxylin and Eosin (H&E) staining using a Leica Auto Stainer XL (Leica

Biosystems). The stained samples were mounted using a Neo-Mount® anhydrous mounting medium (Millipore) and imaged using a Keyence BZ-9000 fluorescence microscope (Keyence Corp.) under bright field.

Immunohistochemical (IHC) staining of cryo-sectioned rat bone samples

Samples were stained to assess the RUNX2 expression using IHC staining, as described in a previous study.[2] In brief, samples were delineated using a hydrophobic barrier pen to minimize the necessary volume of solution during the staining procedure. Followed by washing thrice in 1× tris buffered saline (TBS, pH 7.5, cat. no. T5912, Sigma-Aldrich) for 5 min, 0.1% Protease XXIV (Sigma-Aldrich) was treated for 10 min at 37 °C. Next, cold 1× TBS was used to stop protease activity, and 1× TBS-T (Tween 20 at 0.05%, cat. no. SRE0031, Sigma-Aldrich) was treated twice for 5 min for rinsing. Following that, samples were exposed to a blocking buffer consisting of 1× TBST (0.05% Tween 20, 10% NGS) for 2 h at RT. The primary RUNX2 antibody was diluted in 1× TBS-T buffer and used to treat the samples overnight at 4 °C. The following day, samples were cleansed twice in 1× TBS-T buffer for 15 min. The RUNX2 secondary antibody diluted in 1× TBS-T buffer and Hoechst were utilized to stain the samples for 1.5 h at RT. Finally, the Zeiss LSM880 confocal microscope was used for imaging at 4x and 20x magnification.

Mechanical testing of rat calvarial defects

Mechanical testing was performed using a push-out test, where a probe (4.7 mm dia.) was used, and defects of bone specimens were centrally aligned with respect to a hole on the plate, allowing the probe to pass.[9] Then, uniaxial compression was applied to the bone specimens at a crosshead speed of 0.01 mm/s. The test was terminated when the probe passed through the defects. The stress-strain relationship of regenerated defects was assessed with deformation measurements initiated

by the tester after a 10% compression. Young's modulus of specimens was calculated within a 20 to 40% elastic deformation range using linear curve fitting, ensuring an average R-value exceeding 0.95.[10] Through the push-out test, peak force (N), stiffness (N/mm), and energy (Nmm) were determined.

Boundary BMD of rat calvarial defects, where the push-out probe was applied, were measured to support the mechanical testing results. The difference of diameter between the calvarial defects (5 mm) and the push-out probe (4.7 mm) was 0.3 mm. Therefore, the calvarial defects with a boundary thickness of 3 mm were selected (**Figure S6A**) and measured in BMD.

IOB of bone constructs on a sheep model

A total of six ~3-year-old Dorset sheep (New England Ovis; one male and female sheep allocated for each group) were obtained and housed in the animal facility at Milton S. Hershey Medical Center in accordance with the guidance set by AALAS and IACUC (protocol #00643). Food was withheld for 15 h prior to surgery, while water was provided ad libitum. The sheep were restrained in a dorsal recumbent position, which facilitated the placement of the cephalic catheter without the need for sedation. Anesthesia was induced with intravenous Propofol (4-6 mg/kg) and maintained with isoflurane (1-3%) via inhalation. Continuous monitoring of anesthesia was performed using electrocardiography, pulse oximetry, pulse rate, respiratory rate, end-tidal carbon dioxide ($ETCO_2$), capnography with spirometry, blood gas analysis, and body temperature. Mechanical ventilation was employed to maintain $ETCO_2$ at approximately 35–45 mmHg. Thermal support was provided using a circulating warm water blanket or Bair Hugger. Eye lubricant was applied to prevent corneal desiccation. An orogastric tube was inserted to minimize or prevent ruminal bloat. Oxygen and medical-grade air were administered at 60% and 40%, respectively. Intravenous catheterization was performed using 0.9% sodium chloride, kefzol (30 mg/kg, every 8 h), and

lactated Ringer's solution (60 ml/kg/24 hours; continuous rate infusion). The jugular furrow(s) were clipped and aseptically prepared with chlorhexidine and alcohol, followed by iodine paint. A single or triple lumen central venous catheter was placed in the jugular veins using the modified Seldinger technique and secured with 2-0 Ethilon suture.

Prior to surgery, buprenorphine was administered intravenously at a dosage of 0.005-0.01 mg/kg, and carprofen was injected intravenously at a dosage of 2-4 mg/kg. A 20G over-the-needle catheter was placed in the cephalic vein following the clipping of wool and aseptic preparation of the skin with chlorhexidine and alcohol, repeated three times. The skull was scrubbed three times with chlorhexidine and alcohol, followed by a final application of betadine. The surgical incision site was infiltrated with 1% lidocaine (up to 3 mL) into the subcutaneous tissue. A sagittal scalp incision was made to expose the cranium, specifically the parietal bone.

For sheep calvarial defects, cranial perforators with two compartments (upper compartment: 14 mm diameter and 6 mm thickness; lower compartment: 11 mm diameter and 3 mm thickness) were utilized to create four near critical-sized defects into the parietal bone, two on each side of the sheep skull. An offset in the radius of 1 mm was kept from the periphery to avoid over-extrusion and collision between the nozzle and host bone.

A 6-axis robotic arm (EPSON) was operated for IOB on sheep. Grid structures were coded in Python to generate the Spring Expression Language (SPEL) codes to operate the robotic arm. A reduced robotic arm speed (300 mm/s) was used to ensure the thorough infilling of the substantial defect volume. Counterbored holes consisted of two cylinders on top of one another with diameters of upper and lower cylinders as 13 and 9 mm, respectively, optimized to efficiently fill the bone defect having diameters of upper and lower compartments as 14 and 11 mm. The distance between

the filaments of the grid design was kept such that the adjacent filaments overlapped, and the bioprinted construct filled the defect with 100% infill density.

After the surgery, the sheep were housed in a lamb room under continuous 24/7 care by animal care technicians for the initial two weeks post-operatively. The animals were evaluated using the small ruminant pain assessment scoring system twice daily during the first week following surgery. Catheters were inspected daily by animal care technicians and veterinarians for any signs of infection or inflammation, including redness, swelling, heat, pain, and discharge. Catheter sites were cleaned with chlorhexidine and alcohol three times by a veterinarian every other day, followed by the application of topical triple antibiotic ointment. The necks were then rewrapped with 4 × 4 gauze, vet wrap, and an ace bandage. Once the animals appeared stable and no longer required continuous 24/7 care, they were transferred to regular sheep housing and assessed by a veterinarian at a frequency deemed appropriate by the clinical veterinarian.

After 12 weeks, the animals were euthanized under anesthesia with an overdose of sodium pentobarbital (greater than 150 mg/kg), and the defect regions were explanted. Euthanasia criteria included a veterinary pain score exceeding 12 for 36 h. Additionally, endpoints included a body condition score of less than 2 and/or a 20% weight loss compared to pre-operative weight. Death was confirmed by checking of the heartbeat.

μCT of sheep calvarial defects

Using the SkyScan 1176, μCT images of sheep calvarial defects were captured with a copper and aluminum filter, 18 mm isometric voxels, 90 kV energy, 270 ms exposure, 0.5° rotation degree, and 278 mA intensity. After obtaining a standard curve using the phantom with scanned samples,

two compartments were separately designated. Hard bones above 300 mmHA/ccm were displayed to assess the BV/TV (%), normalized BMD (%), and bone coverage area (%).

Histological analysis of sheep samples

Sheep skulls were segmented into four regions, each containing a single defect. The explants were fixed with 10% neutral buffered formalin (NBF). NBF was changed every 72 h for 1 month. After fixation, the samples were rinsed in running tap water for 6 h and placed in 20% formic acid (Sigma-Aldrich; #695076-500ml). Samples were decalcified in 20% formic acid solution at 30 °C for 3 months. The solution was changed twice a week *(11)*. Decalcification was considered complete when the bone tissue gained elasticity. Upon decalcification, the samples were rinsed in running tap water overnight. After formic acid was removed from the tissue, the decalcified samples were encapsulated in an O.C.T. cryomarix embedding resin and sectioned at -25 °C with 18 μm thickness via the Leica CM1950 cryostat. Subsequently, H&E staining process was performed without heat using the Leica Auto Stainer XL. Followed by mounting the samples using a Xylene Substitute Mountant (Thermo Fisher Scientific), H&E-stained samples were imaged under bright field using the Keyence BZ-9000 fluorescence microscope at 4x and 10x magnifications.

Masson's Trichrome staining (MTS) of rat and sheep samples

The collagen deposition was histomorphometrically evaluated by staining via Masson's Trichrome Staining (MTS) kit (cat. no. HT15-1KT, Sigma-Aldrich), followed by manufacturer's instructions. The stained samples were dehydrated using ethanol with gradually increasing concentrations (50-100%). After mounting the Xylene Substitute Mountant, the staining images were taken using the Keyence BZ-9000 fluorescence microscope under bright field at 4x and 10x magnifications.

Quantitative evaluation was performed using a histology scoring system according to the literature (*8*). Briefly, hard tissue response at bone-scaffold interface was measured from 0 indicating poorly organized tissues to 4 indicating the direct bone-to-implant contact. For pore tissue characterization, the lowest score 0 means the tissue filled with inflammatory cells without bone, while the highest score 4 means the structure with mostly bone. Lastly, bone formation was scored from 0 for 0% bone volume to 4 for 75-100% bone volume with an incremental step of 25%. Two independent reviewers separately assessed three sections per group for rat samples and different depths (edge, periphery, and center) for sheep samples. After reaching a consensus, the scores were averaged and then the resulting averages were used to determine the overall score for the group.

Mechanical testing of sheep calvarial defects

Mechanical testing was performed as per the set-up described before. Samples were tested using a probe (dia.: 10 mm) and the plate with hole (dia.: 12.5 mm) designed for sheep calvarial defects (upper compartment dia.: 14 mm; lower compartment dia.: 11 mm). The uniaxial compression speed was set at 2 mm/min, which was applied until a thorough penetration into the defect was achieved. Subsequently, peak force (N), stiffness (N/mm), and energy (Nmm) were then evaluated.

References

[1] H. Nguyen, D. A. Morgan, M. R. Forwood *Cell Tissue Bank.* **2007,** *8,*81.

[2] K. K. Moncal, H. Gudapati, K. P. Godzik, D. N. Heo, Y. Kang, E. Rizk, D. J. Ravnic, H. Wee, D. F. Pepley, V. Ozbolat *Adv. Funct. Mater.* **2021**, *31,* 2010858.

[3] N. Rajan, J. Habermehl, M.-F. Coté, C. J. Doillon, D. Mantovani *Nat. Protoc.* **2006**, *1,* 275

[4] K. K. Moncal, V. Ozbolat, P. Datta, D. N. Heo, I. T. Ozbolat *J. Mater. Sci. Mater. Med.* **2019**, *30,* 1.

[5] E. M. Noah, J. Chen, X. Jiao, I. Heschel, N. Pallua *Biomaterials* **2002**, *23*, 2855.

[6] I. T. Ozbolat, H. Chen, Y. Yu *Robot. Comput. Integr. Manuf.* **2014**, *30,* 295.

[7] N. Celik, M. H. Kim, M. Yeo, F. Kamal, D. J. Hayes, I. T. Ozbolat *Biofabrication* **2022**, *14,* 044104.

[8] Z. S. Patel, S. Young, Y. Tabata, J. A. Jansen, M. E. K. Wong, A. G. Mikos *Bone* **2008**, *43,* 931.

[9] P. P. Spicer, J. D. Kretlow, S. Young, J. A. Jansen, F. K. Kasper, A. G. Mikos *Nat. Protoc.* **2012**, *7,* 1918.

[10] M. Griffin, Y. Premakumar, A. Seifalian, P. E. Butler, M. Szarko *J. Vis. Exp.* **2016**, e54872.

[11] C. Broomfield, N. Meis, J. Johnson, D. Regan, K. McGilvray, C. Puttlitz *J. Histotechnol.* **2022**, *45,* 29.

Supplementary Tables

Table S1. Bioprinting conditions for in-vitro culture and IOB

Printing parameters	In-vitro	IOB for the rat model	IOB for the sheep model
Set-up temperature (°C)	37 °C	Body temperature (~37 °C)	Body temperature (~39 °C)
Nozzle gauge (diameter)	22 (410 μm inner dia.)	22 (410 μm inner dia.)	20 (910 μm inner dia.)
Printing speed (mm/min)	400	400	300
Extrusion pressure (kPa)	80 to 140	80 to 140	240 to 300
Layer thickness (mm)	0.2	0.5	0.7
Substrate	Glass	Calvarial defect	Calvarial defect

Table S2. Filament size measurements for different bioink conditions

	HT-ink	HC-ink
Cell density (per mL)	**Filament size (target size: 500 μm)**	**Filament size (target size: 500 μm)**
Bioink-only	470.8 ± 47.1 μm	475.5 ± 61.6
1M hADSCs	499.3 ± 42.1 μm	479.4 ± 55.2
5M hADSCs	498.4 ± 45.6 μm	493.2 ± 45.8
10M hADSCs	N/A	509.8 ± 37.4

Table S3. In-vivo study groups for IOB into rat and sheep calvarial defects

Studies	Groups	Endpoint (weeks)	Total # of defects
Rat calvarial defect	Non-treated defect (Empty; control)	8	8
	HT-ink only		
	HT-ink + 1 million (M) hADSCs/mL		
	HT-ink + 5M hADSCs/mL		
	HC-ink only		
	HC-ink + 1M hADSCs/mL		
	HC-ink + 5M hADSCs/mL		
	HC-ink + 10M hADSCs/mL		
Sheep calvarial defect	Non-treated defect (Empty; control)	12	8
	HC-ink only		
	HC-ink + BMP-2		

Table S4. Primer sequences for Col1, RUNX2, Osterix, BSP, and GAPDH.

Primer	Forward primer sequence (5′-3′)	Reverse primer sequence (5′-3′)
Col1	ATG ACT ATG AGT ATG GGG AAG CA	TGG GTC CCT CTG TTA CAC TTT
RUNX2	GGT TAA TCT CCG CAG GTC ACT	CAC TGT GCT GAA GAG GCT GTT
Osterix	CCT CTG CGG GAC TCA ACA AC	AGC CCA TTA GTG CTT GTA AAG G
BSP	AAC GAA GAA AGC GAA GCA GAA	TCT GCC TCT GTG CTG TTG GT
GAPDH	CAC ATG GCC TCC AAG GAG TA	GTA CAT GAC AAG GTG CGG CT

Supplementary Figures

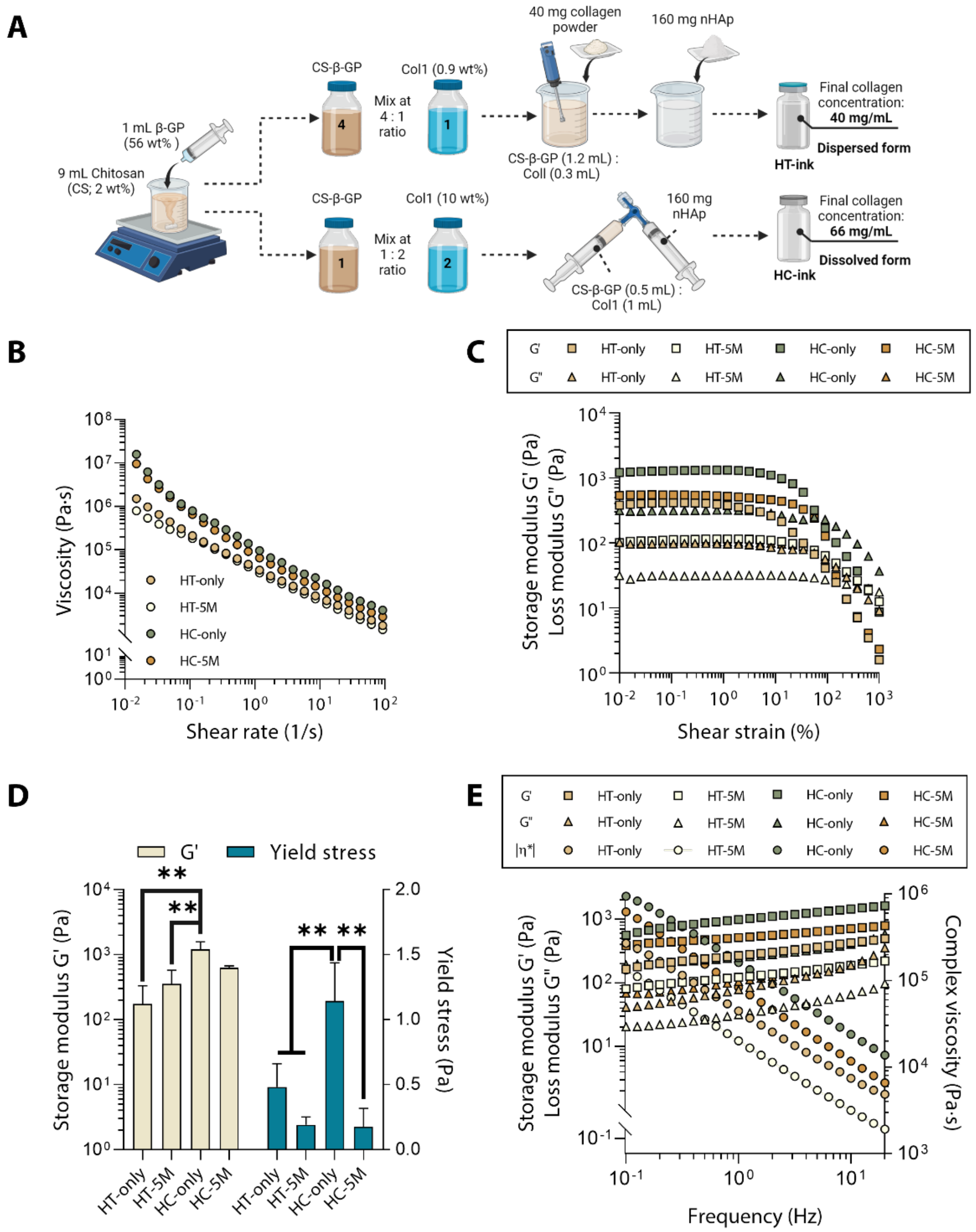

Figure S1. Preparation and evaluation of rheological properties of the HT- and HC-inks. (**A**) Schematic illustration of the process for formulating the HT- and HC-inks. (**B**) Shear thinning test, in which the viscosity curve was obtained using a shear rate ranging from 0.015 to 100 1/s. (**C**) Amplitude sweep measurements, where G' and G'' were examined against strain between 0.01 and

1,000%. (**D**) Comparison of G' (Pa) and yield stress (Pa) for acellular and cell-laden bioinks. (**E**) The frequency sweep test for measuring G', G'', and η^* against frequencies from 0.1 to 20 Hz (n = 3). The asterisk symbol (*) referred to the comparison among all bioink via one-way ANOVA (*$p < 0.05$, **$p < 0.01$).

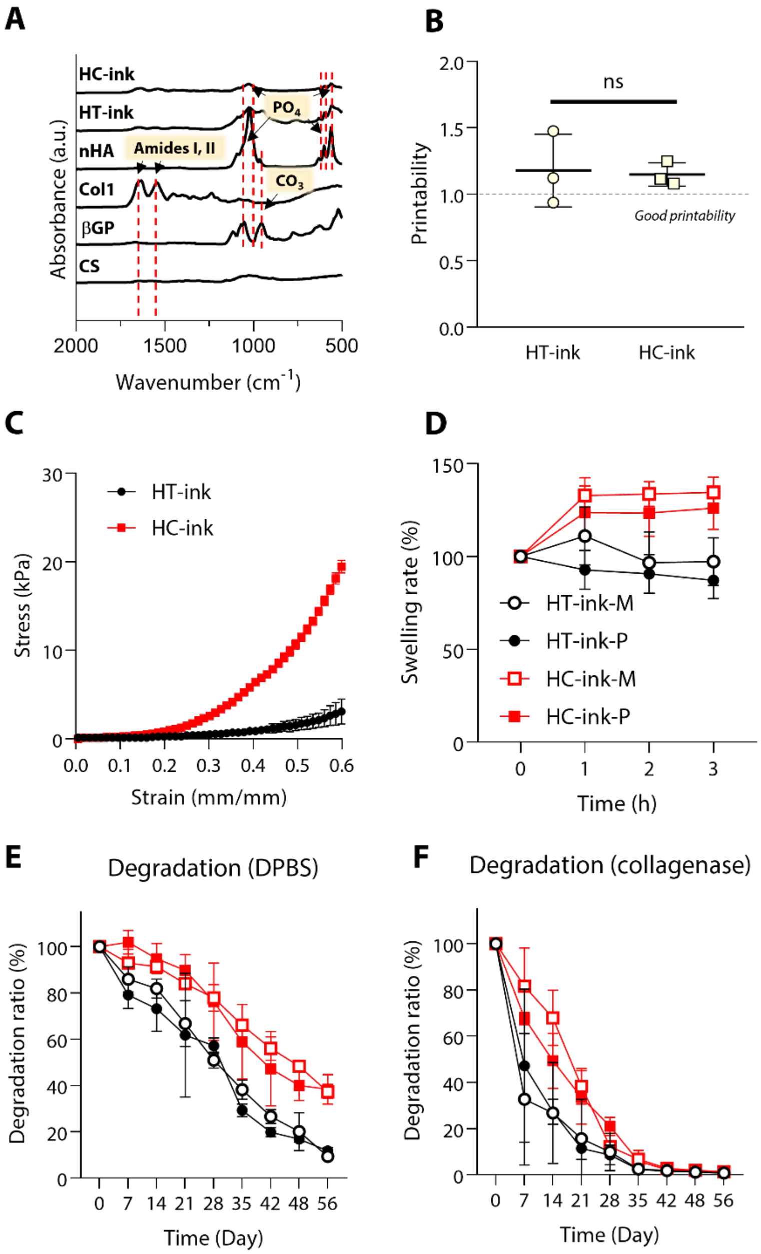

Figure S2. Bioink characterization. (**A**) FTIR of HT-ink, HC-ink, and individual components used to formulate bioinks (n = 3). (**B**) Printability assessment (n = 3). Evaluation of physical properties examined by (**C**) compression test (n = 3), (**D**) swelling rate (n = 6), and (**E**) degradation rate (n = 6).

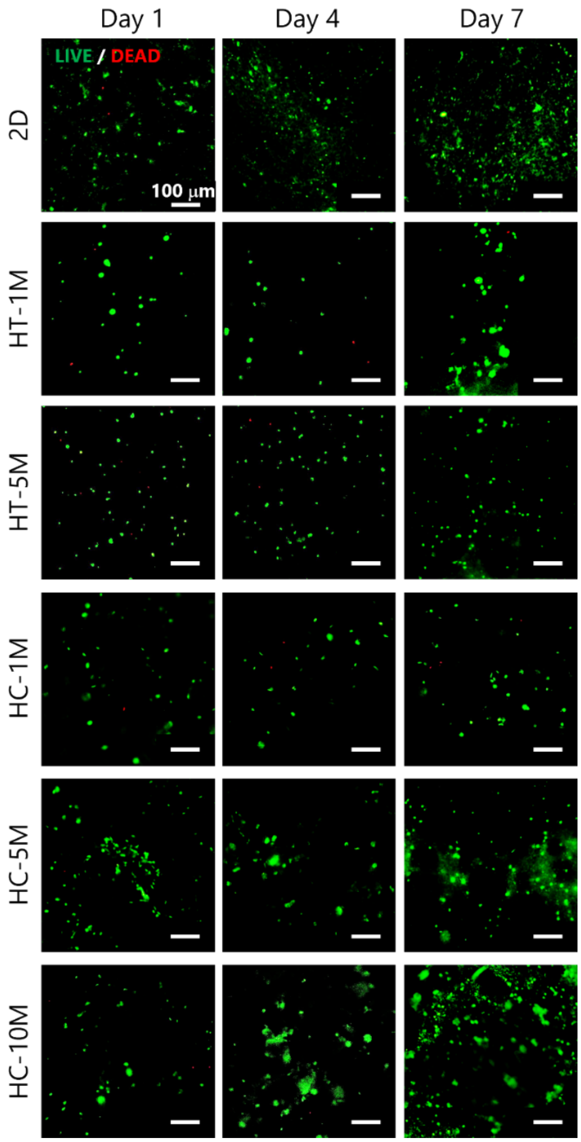

Figure S3. Evaluation of cytocompatibility. Representative LIVE/DEAD imaging for observing live (green) and dead (red) cells to study cytocompatibility of hADSCs in the HT- and HC-ink-laden bioprinted constructs on Days 1, 4, and 7.

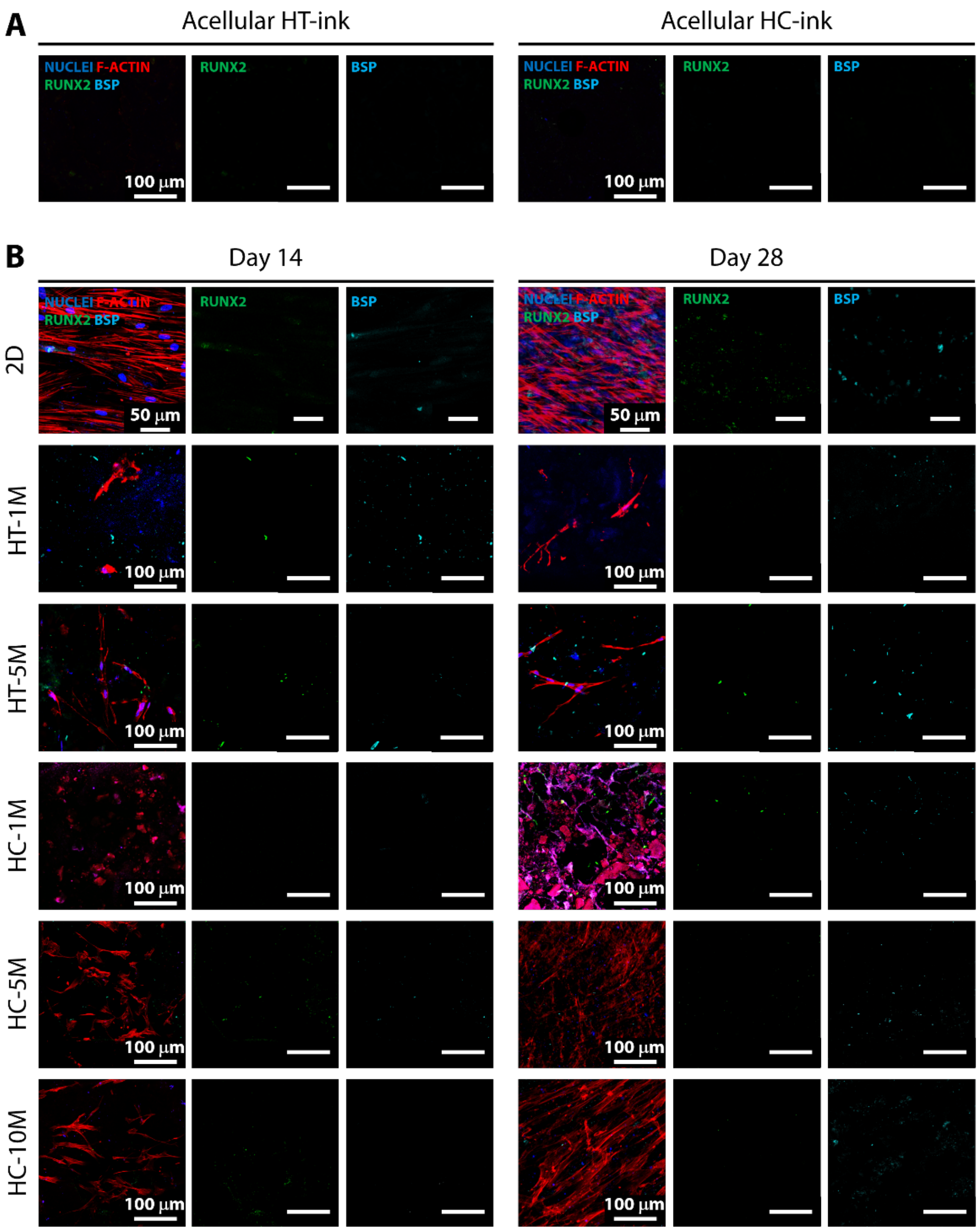

Figure S4. Evaluation of osteogenic behaviors of hADSCs in bioprinted HT- and HC-ink constructs on Days 14 awnd 28. (**A**) Acellular samples stained with DAPI in blue, F-actin in red, RUNX2 in green, and BSP in cyan to verify background staining. (**B**) Immunofluorescence staining of hADSCs portraying RUNX2 in green, BSP in cyan, nuclei in blue, and Phalloidin in red.

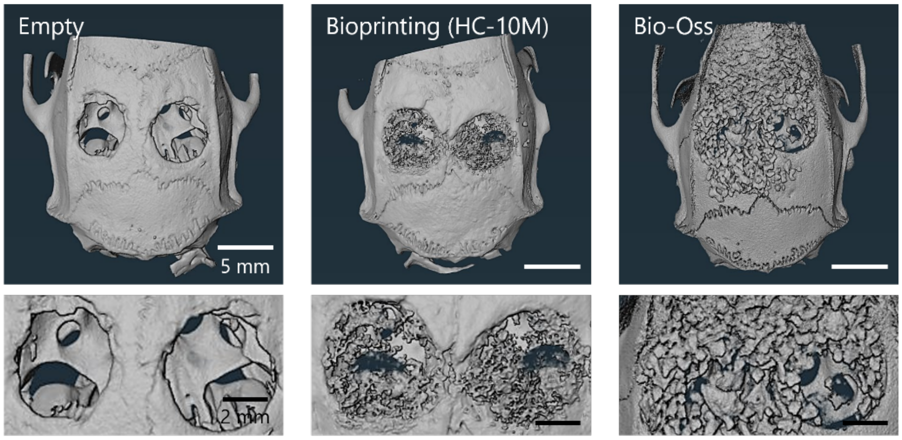

Figure S5. μCT images showcasing the overall morphology of rat calvarial defects at Week 8. Representative images illustrating unfilled defects in the empty group, orthotopic bone regeneration in the bioprinted HC-10M group, and ectopic bone regeneration of the Bio-Oss group (manual loading).

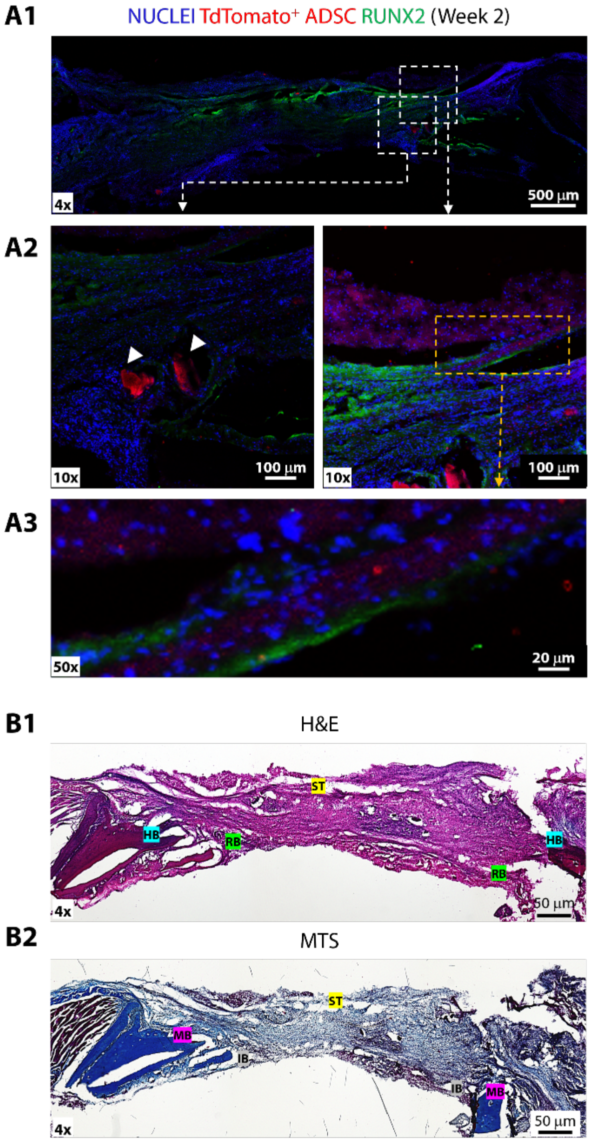

Figure S6. IHC and histomorphometric analysis of rat calvarial defects at Week 2. Fluorescence images of nuclei (blue), tdTomato$^+$ hADSCs (red), and early osteogenic marker RUNX2 (green): (**A1**) the holistic image of the calvarial defect, (**A2**) magnified images of tdTomato$^+$ hADSCs marked by white arrows, and (**A3**) a gradual overlap of tdTomato$^+$ hADSCs (red) and their osteogenic expression of RUNX2 (green). Histological images stained for (**B1**) H&E and (**B2**) MTS, which represent calvarial defects treated with tdTomato$^+$ ADSCs at Week 2.

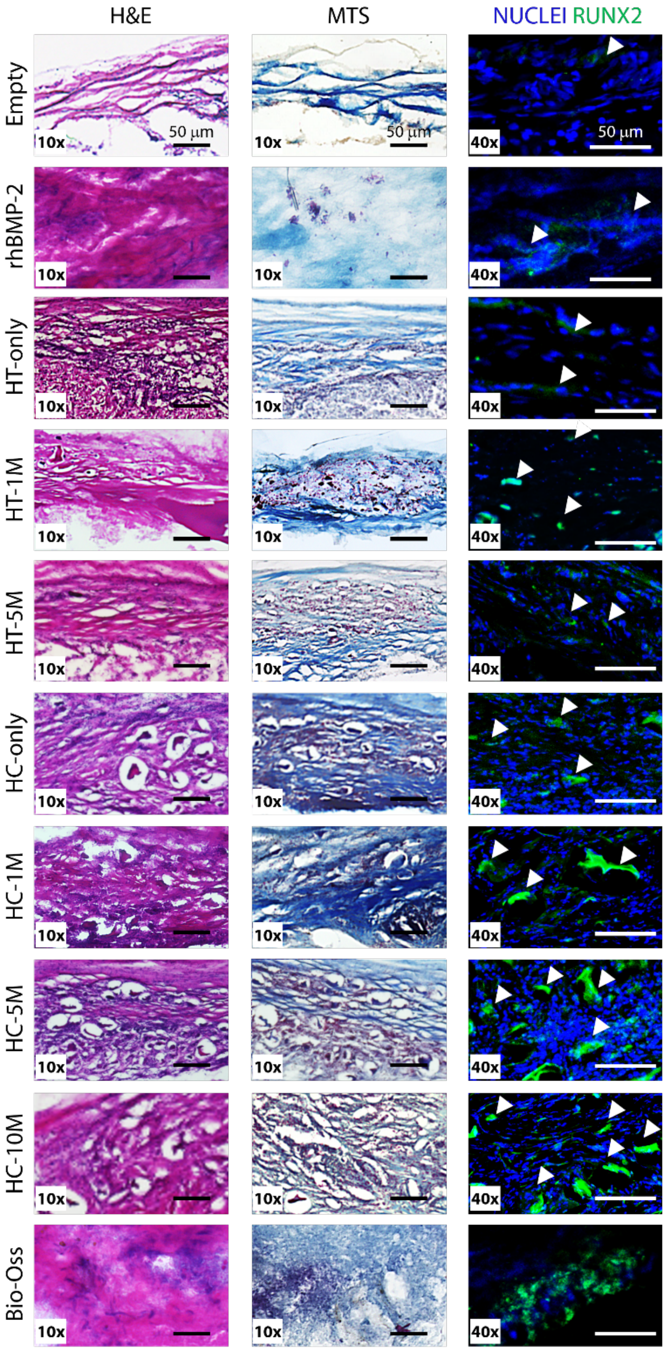

Figure S7. Histomorphometric and IHC analysis of rat calvarial defects at Week 8. Magnified images of H&E (10x), MTS (10x), and IHC (40x) of calvarial defects to represent the regenerated tissues at Week 8. White arrows indicate newly formed bone fragments.

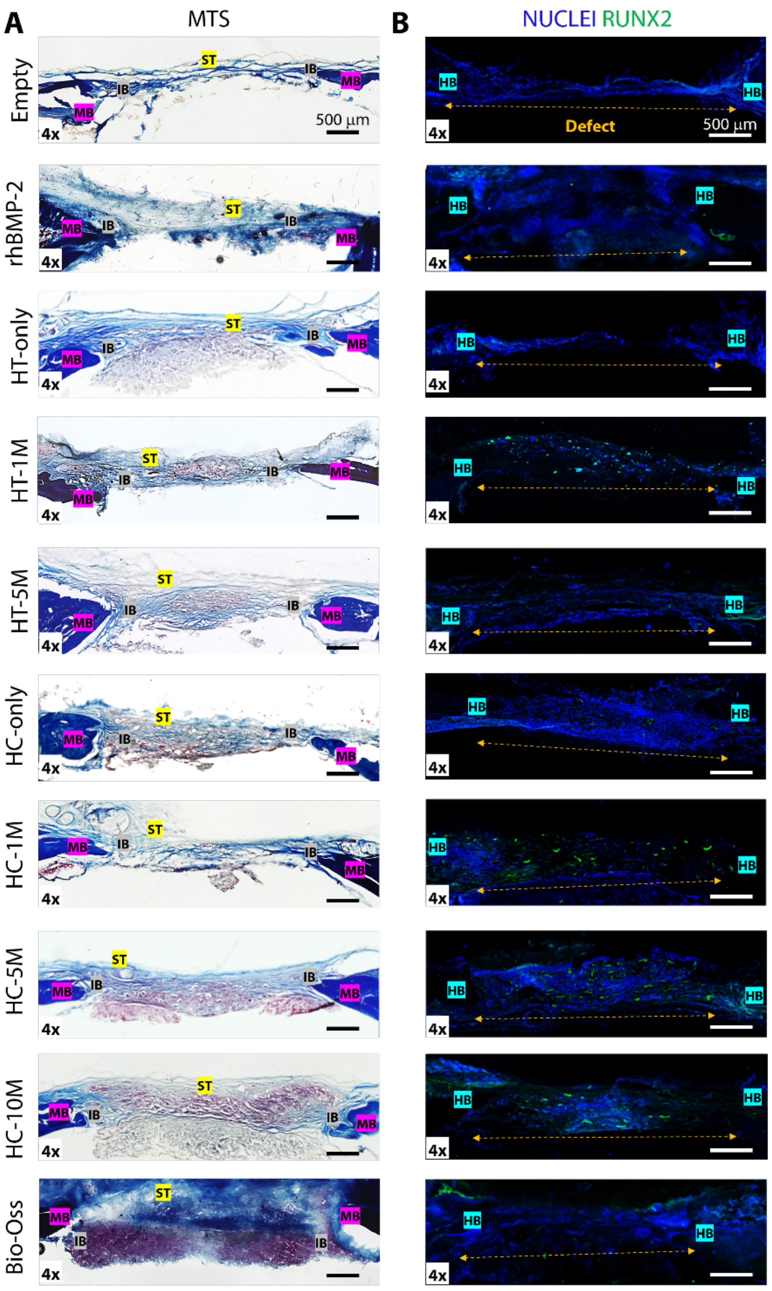

Figure S8. MTS and RUNX2 visualization of rat calvarial defects at Week 8. MTS images demonstrating mature bone (MB), immature bone (IB), and soft tissue (ST). RUNX2 images demonstrating host bone (HB) and the defect regions indicated by yellow arrows. IHC demonstrating RUNX2 (green) counterstained with nuclei (DAPI in blue) to indicate osteogenic effects of the HT- and HC-inks.

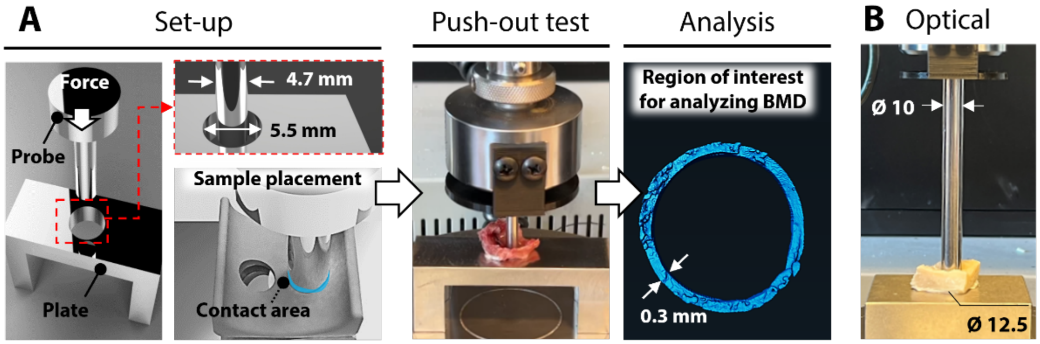

Figure S9. Schematic illustration of mechanical testing of rat and sheep calvarial defect samples. (**A**) Schematic illustration of the mechanical testing setup and the optical image of the push-out test platform for rat calvarial defect samples. The region of interest was selected to analyze normalized BMD, where force was applied ($n = 4$). (**B**) Normalized BMD of edge regions of rat calvarial defects.

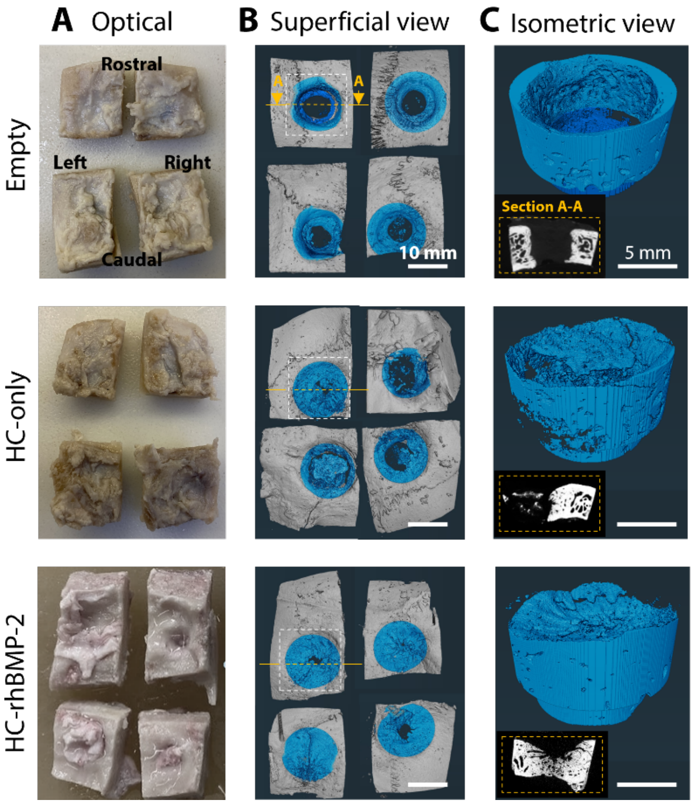

Figure S10. Optical and µCT scanning images after 12 weeks of IOB into sheep calvarial defects. Gross observation demonstrated by (**A**) optical and (**B**) µCT images from the superficial view. (**C**) Isometric view of µCT images demonstrating counterbored structures of the regenerated hard bones (above 300 mmHg/ccm). Cross-section insets of A-A demonstrating bone filling across the defects.

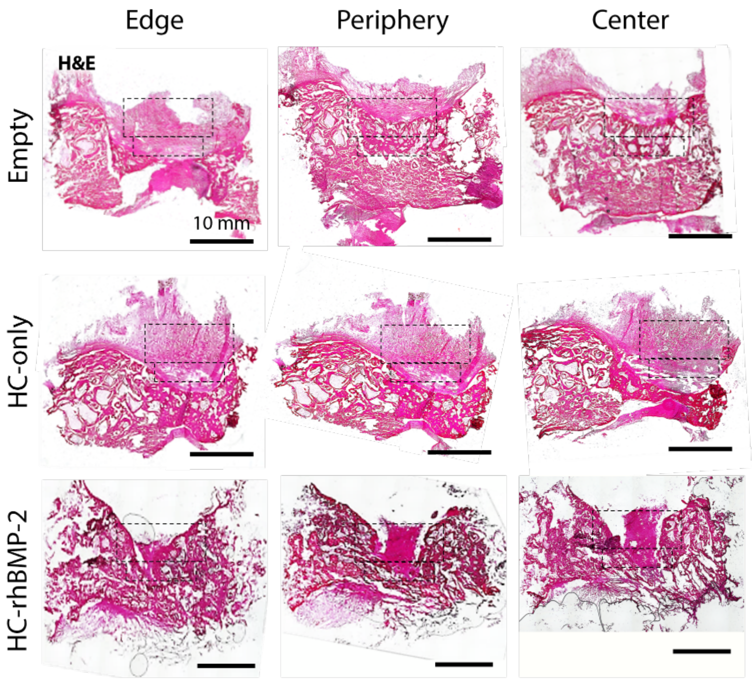

Figure S11. H&E images of sheep calvarial defects at Week 12. Native bone exhibited intense red color staining compared to the regenerated bone.
